# Rapidly evolved genomic regions shape individual language abilities in present-day humans

**DOI:** 10.1101/2025.03.07.641231

**Published:** 2025-03-10

**Authors:** Lucas G Casten, Tanner Koomar, Taylor R Thomas, Jin-Young Koh, Dabney Hofammann, Savantha Thenuwara, Allison Momany, Marlea O’Brien, Jeffrey C Murray, J Bruce Tomblin, Jacob J Michaelson

**Affiliations:** 1 Department of Psychiatry, University of Iowa; 2 Center for Genomic Medicine, Massachusetts General Hospital; 3 Department of Otorhinolaryngology-Head and Neck Surgery, University of Maryland; 4 Carver College of Medicine, University of Iowa; 5 Stead Family Department of Pediatrics, University of Iowa; 6 Department of Communication Science and Disorders, University of Iowa

## Abstract

Minor genetic changes have produced profound differences in cognitive abilities between humans and our closest relatives, particularly in language. Despite decades of research, ranging from single-gene studies to broader evolutionary analyses[1, 2, 3, 4, 5], key questions about the genomic foundations of human language have persisted, including which sequences are involved, how they evolved, and whether similar changes occur in other vocal learning species. Here we provide the first evidence directly linking rapidly evolved genomic regions to language abilities in contemporary humans. Through extensive analysis of 65 million years of evolutionary events in over 30,000 individuals, we demonstrate that Human Ancestor Quickly Evolved Regions (HAQERs)[5] - sequences that rapidly accumulated mutations after the human-chimpanzee split - specifically influence language but not general cognition. These regions evolved to shape language development by altering binding of Forkhead domain transcription factors, including *FOXP2*. Strikingly, language-associated HAQER variants show higher prevalence in Neanderthals than modern humans, have been stable throughout recent human history, and show evidence of convergent evolution across other mammalian vocal learners. An unexpected pattern of balancing selection acting on these apparently beneficial alleles is explained by their pleiotropic effects on prenatal brain development contributing to birth complications, reflecting an evolutionary trade-off between language capability and reproductive fitness. By developing the Evolution Stratified-Polygenic Score analysis, we show that language capabilities likely emerged before the human-Neanderthal split - far earlier than previously thought[3, 6, 7]. Our findings establish the first direct link between ancient genomic divergence and present-day variation in language abilities, while revealing how evolutionary constraints continue to shape human cognitive development.

## 2 Main

The human genome differs from that of our closest living relatives by only 1–5%[8, 9, 10], yet this modest genetic divergence underlies profound differences in cognitive functions, particularly language. Understanding how such minor genetic changes gave rise to complex human abilities has been a central challenge in evolutionary genetics. Despite decades of research, we still lack a clear understanding of the genomic basis of human language ability – a trait that fundamentally shapes human cognition and culture.

The search for genetic foundations of human language accelerated with the discovery that mutations in *FOXP2* can cause speech and language disorders[1, 2]. While initially heralded as “the language gene,” *FOXP2* ‘s role in more typical variation in language ability proved to be elusive[11, 12]. Subsequent research shifted focus to numerous non-coding elements distributed throughout the genome[13, 14, 15, 16, 17]. This distributed regulatory model better captured the complexity of language but left open questions about evolutionary origins and how these elements influence language ability when perturbed.

Here, we provide evidence bridging these perspectives through systematic analysis of 65 million years worth of primate and human evolutionary events. We find one class of human specific genetic regions to be particularly important to human language, Human Ancestor Quickly Evolved Regions (HAQERs)[5] – sequences that began accumulating mutations at an unusually high rate after the human-chimpanzee ancestral split. Despite comprising a small fraction of the genome, HAQERs show robust and specific associations with core language ability, but not general cognition, across multiple large cohorts. Strikingly, we find these regions are under selection for increased affinity to Forkhead box transcription factor binding motifs (including *FOXP2* ), with this motif integrity directly linked to language ability in a sample of modern humans. This finding provides a theoretical framework that explains both the impact of rare coding variants in genes like *FOXP2* and the importance of distributed regulatory variation in shaping individual differences in language ability.

Our discovery emerged from a multi-modal study combining evolutionary genomics with language phenotyping and whole genome sequencing in present-day humans. We analyzed 350 children followed longitudinally with extensive language testing, validating our findings in larger cohorts including the SPARK study (N > 30,000) and the ABCD study (N > 5,000)[18, 19]. We further examined selective pressures acting on language and general cognition using ancient DNA from early humans, Neanderthals, and Denisovans (Allen Ancient DNA Resource, N > 3,000)[20]. This revealed that language-promoting variation in HAQERs has remained stable across humans throughout the past 20,000 years – we find a possible explanation for the apparent lack of positive selection as these variants have pleiotropic effects that increased fetal brain growth as well as birth complications. Finally, we investigated convergent evolution of HAQER-like sequences through analysis of 170 non-primate species[21], finding additional evidence for the key role of these regulatory sequences in vocal learning.

Our results demonstrate for the first time how ancient genomic changes directly influence individual variation in modern language abilities, reveal the central role of Forkhead box transcription factors in language evolution, and provide empirical support for a complex, ancient, and polygenic model of language evolution. By connecting evolutionary genomics with functional impacts on modern human cognition, our study provides triangulated support for the role of HAQERs in language development and opens new avenues for investigating the genetic foundations of human-specific traits.

## 3 Dimensions of language ability

**Figure 2:**
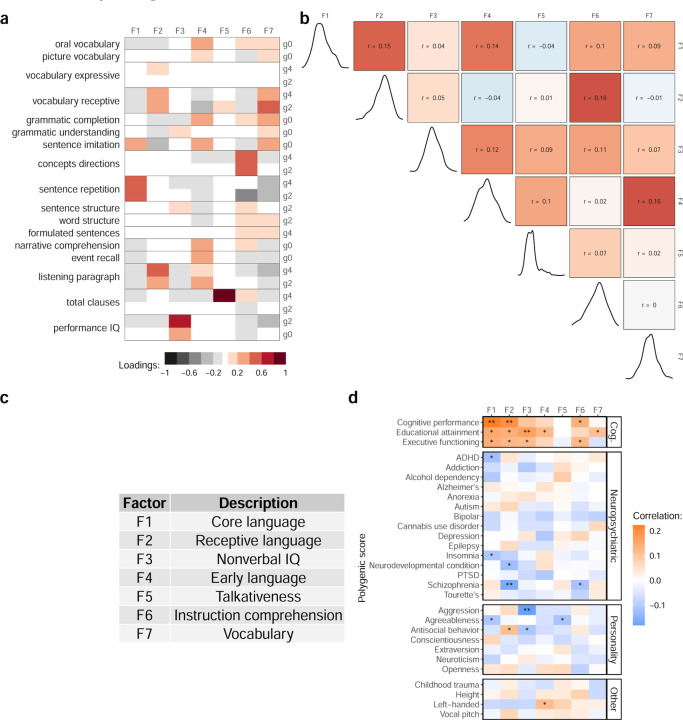
Factor loadings and genetic associations **a** Loadings of cognitive and language assessments onto the seven language factors. g0 = Kindergarten (age 5–6), g2 = 2nd grade (age 7–8), g4 = 4th grade (age 9–10). **b** Pearson correlations for language factors (upper triangle) and distribution of each factor (diagonal). **c** Interpretations of the language factors based on their loadings. **d** Pearson correlations for each factor with genome-wide PGS. ** indicates FDR adjusted p-value *<* 0.05 and * indicates unadjusted p-value *<* 0.05.

To quantify dimensions of developmental language abilities, we analyzed 17 longitudinal cognitive and language assessments administered from kindergarten through 4th grade for 350 children sampled from a community-based cohort[22], which we refer to as the “EpiSLI” cohort. This analysis revealed seven factors representing distinct aspects of language ability (Figure 2a). The first factor (F1), primarily driven by sentence repetition scores, represents “core language” ability. Sentence repetition strongly indicates overall language capacity, making F1 a key measure of general language competence[23, 24]. The second factor (F2) relates to receptive vocabulary and listening comprehension, covering broad receptive language skills. The third factor (F3) specifically reflects nonverbal IQ, aligning with performance IQ at both kindergarten and 2nd grade. Factor F4 captures pre-literacy language skills, incorporating all kindergarten scores except performance IQ. Its slight correlation to F1 and F2 (r = 0.13 and 0.12), but not F3, suggests specificity to language (Figure 2b). Factor F5, which we call “talkativeness,” mainly reflects the number of clauses produced in a narrative task. Factor F6, based on a comprehension of concepts and directions assessment, indexes mastery of directive language (i.e., task-based instructions). Factor F7 spans a variety of assessments, with specific loading on vocabulary and grammar-related tasks, suggesting a broad, crystallized knowledge of language.

Most of our preliminary investigation of these factors suggested that Factors 1, 2, and 3 carried the most genetic association signal (Figure 2c, Supplementary Table 3), so these were the focus of the work we present here. We also find pervasive associations with F1-F3 and measures of mental health in our sample (N = 241, Supplementary Table 2).

## 4 Linking evolution to individual differences in language ability with ES-PGS analysis

To investigate the genetic basis of language ability, we developed a novel analytical approach: Evolution Stratified Polygenic Score (ES-PGS) analysis. This method allows for systematic examination of how genetic variants from different evolutionary periods contribute to a trait by partitioning polygenic scores based on the evolutionary origin of the DNA sequence. The ES-PGS method extends traditional polygenic score analysis by comparing reduced and full statistical models, introducing an additional term that captures the contribution of specific evolutionary annotations to language ability. ES-PGS shares similarities with “partitioned heritability” and “pathway-based polygenic scores”[25, 26, 27, 28], which attribute trait heritability to specific genomic regions. The key difference from partitioned heritability methods is that ES-PGS uses individual-level data for predictions, enabling direct association testing in smaller but more deeply phenotyped cohorts. More information about the ES-PGS method and implementation can be found in the Methods section and our code repository (https://github.com/lucasgcasten/language_evolution).

Our genome-wide analysis identified significant associations between the polygenic score (PGS) for cognitive performance[29] (CP-PGS) with both core language (*r* = 0.22, FDR adjusted p-value = 0.001, F1) and receptive language ability (*r* = 0.19, FDR adjusted p-value = 0.01, F2) but not for nonverbal IQ (*r* = 0.1, FDR adjusted p-value = 0.33, F3, Figure 2d) in our EpiSLI sample. We then applied our ES-PGS method to the CP-PGS to determine whether the genomic regions influencing core language (F1) and receptive language (F2) originate from deeply conserved sequences (primate conserved sequence regions), or more recent additions to the human genome (human-Neanderthal divergent regions). By comparing contributions from 11 established evolutionary annotations spanning approximately 65 million years of evolution - from ancient primate-conserved regions to sequences under selection in the past few thousand years — we traced the evolutionary origins of language-related genomic elements.[21, 30, 31, 5, 32, 33, 34, 35].

**Figure 3:**
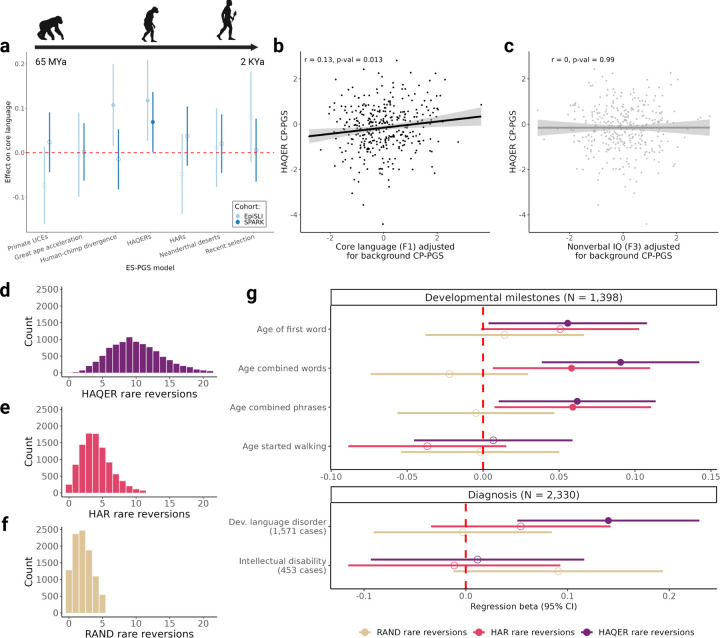
HAQERs are associated with language ability and not nonverbal IQ **a** Comparison of evolutionary events on core language ability in EpiSLI and SPARK. Points represent the *β* provided from the ES-PGS models for each evolutionary annotation, while the ranges represent the 95% confidence interval. Solid points indicate p-value *<* 0.05. **b** Scatterplot of core language scores (F1) with HAQER CP-PGS after adjusting for the background PGS in the EpiSLI sample. **c** Scatterplot of nonverbal IQ scores (F3) with HAQER CP-PGS after adjusting for the background PGS in the EpiSLI sample. **d-f** Distributions of rare reversions counts from the SPARK whole genome sequencing data within 10Kb of the following regions: HAQERs (d), HARs (e), and random sequence (RAND, f). **g** Effects of rare reversions across HAQERs, HARs, and RAND on language related phenotypes in SPARK autism cases. Line ranges represent 95% confidence intervals from logistic and linear regression models. A positive *β* indicates delayed developmental age or higher likelihood of the diagnosis as reversions increase.

As expected, CP-PGS in primate ultra-conserved elements had no association with language scores (ES-PGS model *β* = −0.074, p-value = 0.1). Also in line with expectations, CP-PGS in genes that are differentially regulated between humans and our closest living primate relative (human-chimp divergent genes) is significantly positively correlated with core language ability (ES-PGS model *β* = 0.107, p-value = 0.023), Figure 3a. This finding confirms that human-specific genetic sequence influences language abilities in contemporary humans, and this sequence evolved after the human-chimpanzee split (approximately 6–8 million years ago).

The most compelling finding emerged from the analysis of Human Ancestor Quickly Evolved Regions (HAQERs). Although they represent a small proportion of the human genome, HAQER-associated polygenic scores revealed robust and specific associations with language capabilities. Specifically, CP-PGS in HAQERs showed significant correlations with core language ability (ES-PGS model *β* = 0.117, p-value = 0.012, Figures 3a-b) and receptive language skills (ES-PGS model *β* = 0.098, p-value = 0.022), and without any association with nonverbal intelligence (ES-PGS model *β* = 0.0007, p-value = 0.99, Figure 3c). HAQERs, which began evolving approximately 6–8 million years ago – after the human-chimpanzee split but before the human-Neanderthal divergence – represent largely non-coding sequences that have rapidly acquired regulatory functions in humans. The genomic specificity of these regions is particularly striking. While the background CP-PGS utilized nearly 300,000 independent SNPs (single nucleotide polymorphisms) and explained 3.47% of variance in core language ability, the addition of HAQER CP-PGS (comprising only 1,350 independent SNPs) increased the explained variance to 5.22%, suggesting that HAQER SNPs carry on average 112 times more explanatory power for language than SNPs elsewhere in the genome.

Notably, we found no comparable signal from Human Accelerated Regions (HARs, *β* = −0.048, p-value = 0.29), which are deeply conserved regulatory elements that acquired human-specific changes[4, 32]. This distinction suggests that human language ability emerged disproportionately through novel regulatory innovations (HAQERs), rather than modifications to existing functional elements (HARs). The specific association between HAQERs and language factors (F1 and F2), but not nonverbal IQ (F3), reveals a distinct evolutionary trajectory for verbal abilities compared to general cognition.

## 5 Confirming HAQERs impact on language ability across the lifespan

Next, we validated our finding that HAQERs influence human language by examining the effects of both common and rare variants in these regions with the SPARK autism dataset[18]. The considerable heterogeneity in language abilities observed in autism populations makes SPARK an especially informative dataset for validating language-related genetic discoveries, as this variation enhances our ability to detect associations across the full spectrum of language abilities[36, 37]. Although SPARK is not as deeply phenotyped for language traits as our discovery sample, we have several meaningful language indices. First, we conducted a recontact study in more than 1,000 adults with and without autism (N = 917 with genetic data), in which we asked participants to complete an online battery of language tasks [38]. The sample has a “core language” factor that is comparable to the core language factor we find in EpiSLI (F1), it loads primarily onto sentence repetition, comes from a similar exploratory factor analysis, and is significantly correlated with CP-PGS (*r* = 0.2, p-value = 1.1×10^−8^). Additionally, in a much larger sample of autistic children in SPARK we have parent-reported developmental language disorder (N = 30,203) and verbal IQ from clinical records (N = 1,462). In all of these traits, we found that the HAQER partition of CP-PGS was significantly associated with language outcomes (Figure 3a, Supplementary Table 6). Further, to investigate specificity of HAQER-linked CP-PGS to language, we tested for association with measures of nonverbal IQ (N = 1,547) and intellectual disability (N = 30,203). There was no association between the HAQER partition of CP-PGS and the non-language related measures of cognitive function, indicating genetic variation in HAQERs specifically influences language and not other aspects of cognition (all statistics can be found in Supplementary Table 6).

Additionally, we analyzed the effects of rare genetic variation in HAQERs on cognitive traits with whole genome sequencing data from SPARK. We found that the vast majority of individuals carry rare “reversions” in and around HAQERs (variants with an allele frequency < 1% and “revert” to the human-chimp ancestor allele), indicating these regions remain highly polymorphic in modern human populations when compared to HARs and random sequences (Figure 3d-f). Whole genome sequencing data, developmental milestone information, and diagnosis information were available for more than 2,000 individuals with autism. Individuals with more rare reversions in HAQERs have delayed language development and are more likely to have developmental language disorders (Figure 3g, Supplementary Table 7).

Further supporting the specificity of HAQERs to language ability, we found that rare reversions in these regions showed no association with age started walking or intellectual disability (Figure 3g). To show that the effect is due to HAQERs and not any reversion across the genome, we also analyzed the effects of rare reversions in HARs[4] and random (RAND) non-coding sequence matched to HAQERs[5]. There was no association between rare reversions in random sequence with any of our phenotypes. We did find that rare reversions in HARs were associated with two of the four language phenotypes: age first combined words and age first combined phrases; but not with age of first word or developmental language disorder.

Taken together, our discovery and replication samples provide consistent and compelling evidence that genetic variation in HAQERs is associated with language traits, and not nonverbal IQ, in contemporary humans. In contrast, we do not find consistent evidence that genetic variation in HARs is associated with language, though rare reversions in HARs appear to impact some language development milestones independently of HAQERs.

## 6 Forkhead motifs in HAQERs are under selection and improve language

To investigate the molecular mechanisms underlying HAQERs’ role in language development, we analyzed how two classes of rare variants affect transcription factor binding sites (TFBS) in the EpiSLI cohort: (1) rare (minor allele frequency < 1%) human-chimp ancestral allele reversions and (2) other rare variants. Using position weight matrices (PWMs), we quantified how these variants alter TFBS motif scores (i.e., predicted TF binding affinity). By comparing the effects of reversions with other rare variants, we could detect signatures of human-chimp divergent selection on TF motif scores.

**Figure 4:**
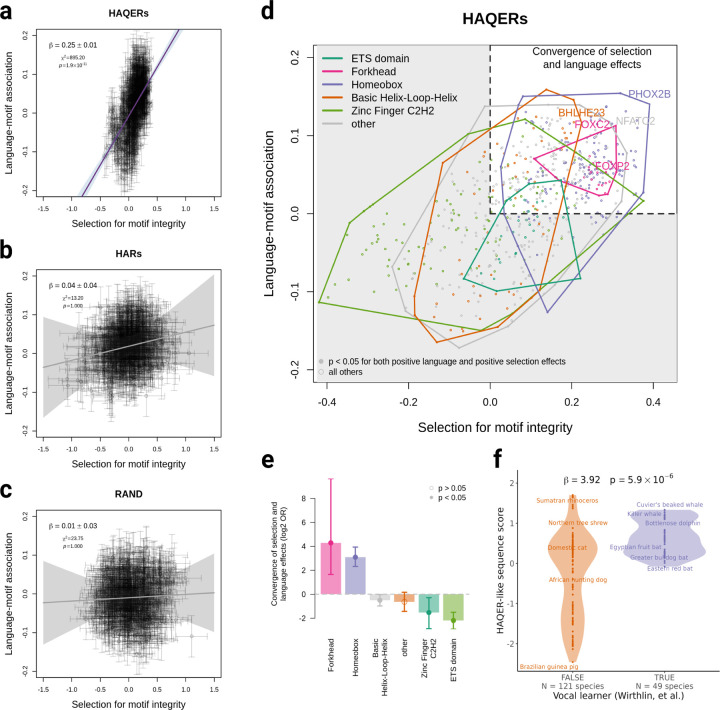
HAQERs show coupled evolutionary and functional effects on language-relevant transcription factor binding sites **a-c** Relationship between selection for transcription factor motif integrity (x-axis) and motif association with language ability (y-axis) in (a) HAQERs, (b) HARs, and (c) random genomic regions. Each point represents one transcription factor motif. Error bars indicate ±1 standard error. Purple line (or gray for non-significant fits) shows York regression fit with 95% confidence interval (shaded); regression coefficient (*β*), chi-squared statistic (*χ*^2^), and p-values are shown. **d** Detailed view of motif effects in HAQERs colored by transcription factor family. Solid points indicate motifs with p < 0.05 for both positive selection and positive language association. Colored polygons show convex hulls for each transcription factor family. Several key Forkhead family members are labeled. Dashed lines at x = 0 and y = 0 define quadrants. **e** Enrichment analysis of transcription factor families for concordant positive selection and language effects, shown as log2 odds ratios. Error bars indicate 95% confidence intervals. Solid points indicate p < 0.05. **f** HAQER sequence similarity scores in vocal learning (blue) versus non-vocal learning (orange) mammals. Violin plots show score distributions, with individual species are indicated by points. Phylogenetic logistic regression coefficient (*β*) and p-value are shown.

In HAQERs, our estimates of transcription factor motif selection showed a significant correlation with those motifs’ estimated impact on individual core language ability (*β* = 0.25, p-value = 1.9×10^−11^, Figure 4a), suggesting humans evolved to increase TF binding in these regions and this binding is associated with better core language ability (F1). In contrast, regions under sequence conservation (HARs, *β* = 0.04, p-value = 1, Figure 4b) or neutral evolution (RAND sequences, *β* = 0.01, p-value = 1, Figure 4c) showed no relationship between motif integrity selection and language ability. This highlights HAQERs’ unique and systematic selection for regulatory function during hominin evolution.

Analysis of transcription factor binding in HAQERs revealed a striking enrichment of Forkhead box TFs in the upper right quadrant of the motif selection-language association space (Figure 4d), with every member of the Forkhead box TF family in this quadrant. This quadrant represents TFs showing both positive selection for binding site integrity and positive effects on core language ability (i.e., increased binding in HAQERs is associated with better language), the Forkhead box family displaying the strongest enrichment among all TF families (odds ratio = 19.53, p-value = 1.3×10^−5^; Figure 4e, Supplementary Table 16). *FOXC2* demonstrated particularly strong signals of human-gained binding affinity (*β* = 0.31, p-value = 4.2 × 10^−12^) and association with language ability (*β* = 0.11, p-value = 0.035). *FOXP2* showed a similar directional trend but did not meet significance criteria (human-gained binding affinity *β* = 0.24, p-value = 7.7 × 10^−8^, but a p-value > 0.05 in the language association test). Our data reveals that binding of the Forkhead box family within HAQERs played a crucial role in the evolution of human language.

## 7 Selective pressures acting on language and general cognition

**Figure 5:**
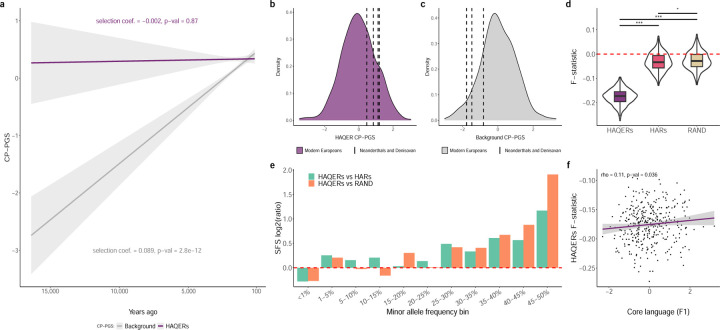
Selective pressures acting on language alleles in HAQERs **a** Polygenic selection of HAQER and background CP-PGS, correlating sample age with CP-PGS in ancient west Eurasians from the AADR. **b-c** Distribution of HAQER CP-PGS (b) or background CP-PGS (c) in modern Europeans (N = 503 individuals from the 1000 Genomes dataset), with black dotted lines indicating the PGS in the four Neanderthal and Denisovan genomes. **d** Comparison of F-statistics across HAQERs, HARs, and random sequences (RAND). F-statistics measure heterozygosity enrichment, with lower values indicating more heterozygosity than expected. “***” is used to indicate statistical significance (p-value < 0.001) based on t-test comparisons between each pair of regions. **e** Site Frequency Spectrum (SFS) comparison between HAQERs, HARs, and random sequences (RAND). x-axis represent minor allele frequency bins, y-axis is the log2(ratio) comparing HAQERs to the other sequence types. Positive log2(ratios) indicate that HAQERs have proportionally more variants in that allele frequency bin compared to the other sequence type. **f** Correlation between core language ability (F1, x-axis) with F-statistics in HAQERs (y-axis).

Having established HAQERs’ role in human language evolution, we next examined how language-related genetic variants have been selected for throughout the past 20,000 years of human history using the Allen Ancient DNA Resource (AADR)[20]. The AADR is the largest genotyped collection of ancient humans, providing harmonized genotype and metadata for each sample (like radiocarbon dating based sample ages). We identified ancient west Eurasians, then correlated their HAQER CP-PGS and the background CP-PGS with sample age (N = 3,244 individuals with remains dated between 18,775 to 150 years ago passing quality control). We see that general cognition (background CP-PGS) has been subject to positive selection and has increased substantially over time (selection coefficient = 0.089, p-value = 2.8 × 10^−12^), Figure 5c. Unexpectedly, we found that HAQER CP-PGS has been stable throughout human history - meaning ancient and modern humans carry similar numbers of language-related alleles in HAQERs (selection coefficient = −0.002, p-value = 0.87).

The presence of HAQERs in archaic humans provided a unique opportunity to examine genetic potentiators of cognitive traits across human species. To do this, we computed HAQER CP-PGS and background CP-PGS in archaic humans and compared them to modern humans. Remarkably, all four high-coverage archaic human genomes (three Neanderthals and one Denisovan) showed elevated HAQER CP-PGS (mean z-score = 0.91), while having reduced background CP-PGS (mean z-score = −1.45, 5a-b, Supplementary Table 12). The elevated HAQER CP-PGS observed in archaic humans remains consistent even after accounting for population structure and limiting our analysis to HAQER SNPs directly genotyped across all archaic samples, indicating this result is unlikely to be a technical artifact. This unexpected pattern suggests that our ancient relatives had an elevated genetic predisposition for language abilities despite lower scores for general cognition, challenging traditional views of archaic human capabilities.

The striking stability of the HAQER CP-PGS throughout human evolutionary history, potentially predating the human-Neanderthal split, led us to hypothesize that HAQERs have been maintained through balancing selection. To test this hypothesis, we conducted multiple population genetic analyses in the EpiSLI sample comparing HAQERs to both HARs and matched random genomic sequences (RAND). First, we observed an enrichment of intermediate frequency variants (MAF 10–50%) in HAQERs - a characteristic signature of balancing selection (Figure 5e). This enrichment pattern indicates a “heterozygote advantage”, where selective pressures are actively maintaining genetic diversity in these regions rather than driving language-beneficial alleles to fixation. To directly evaluate a heterozygote advantage in HAQERs, we calculated F-statistics across these regions, which quantify the deviation between observed and expected heterozygosity. HAQERs exhibited significantly lower F-statistics compared to both HARs (t-statistic = −55.3, p-value = 8 × 10^−254^) and random sequence (t-statistic = −57.9, p-value = 1.8 × 10^−265^), indicating excess heterozygosity in HAQERs (Figure 5d). Intriguingly, we discovered that individuals with higher F-statistics in HAQERs—those possessing more homozygous genotypes across these regions—displayed significantly better language abilities (rho = 0.11, p-value = 0.036, Figure 5f). This apparent paradox can be resolved by a model of antagonistic pleiotropy, where homozygosity for certain HAQER variants enhances language ability but potentially decreases reproductive fitness. These findings collectively support a balancing selection model in which a heterozygote advantage for reproductive outcomes constrains the fixation of language-enhancing alleles in HAQERs, maintaining these variants at intermediate frequencies throughout human history.

## 8 HAQERs influence birth complications and prenatal neurodevelopment

The well-established neurodevelopmental functions of HAQERs guided our hypothesis about balancing selection in these regions[5]. We hypothesized that while variants in HAQERs can benefit language ability, these same variants likely created reproductive challenges through adverse effects on fetal development, possibly through birth complications and increased energy requirements, thus preventing their fixation despite their cognitive advantages. To test this hypothesis, we examined links between HAQERs and prenatal development in modern humans with the ABCD sample[19]. We conducted a within-family analysis, comparing ES-PGS between siblings and their differences in birth outcomes, allowing us to more rigorously account for environmental effects than typical population scale analyses. We found that HAQER CP-PGS influences birth weight and birth complications within families (N > 500 sibling pairs from ABCD). The difference between sibling birth weight was positively associated with their difference in HAQER CP-PGS (ES-PGS *β* = 1.62, p-value = 0.03), meaning the sibling born heavier carries more HAQER CP-PGS (Figure S1f). We did not observe an association with difference in birth weight and background CP-PGS (ES-PGS *β* = −0.17, p-value = 0.82, Figure S1e). We also examined 47 of these sibling pairs where only one was born via c-section (Figure S1h). The siblings born by c-section had higher HAQER CP-PGS (t-statistic = 2.06, p-value = 0.045), and no difference in their background CP-PGS (t-statistic = 0.02, p-value = 0.88, Figure S1c-d).

We sought to further expand our understanding of HAQERs neurodevelopmental influences by examining their regulatory effects during prenatal and postnatal periods. By intersecting HAQER regions with single-cell quantitative trait loci (scQTLs) from developing midbrain neurons, we found HAQERs are significantly enriched for variants influencing prenatal brain gene expression. Our analysis revealed HAQERs are enriched for regulatory elements across all observed prenatal timepoints and almost all cell types, suggesting a broad neurodevelopmental impact (Figure S1a). Critically, these regions showed no enrichment in adult brain scQTLs, indicating their influence occurs primarily during prenatal development (Figure S1b).

To further test evidence of a HAQERs influence brain structure prenatally, we associated HAQER CP-PGS intracranial volume and intracranial growth phenotypes using a large independent sample of adolescents (ABCD). Finding that HAQER CP-PGS was predictive of total intracranial volume, even after accounting for body size (ES-PGS *β* = 0.03, p-value = 0.02, N = 5,274 unrelated individuals, Figure S1g). HAQERs were not predictive of intracranial growth in adolescence, supporting that HAQERs influence brain development prenatally (ES-PGS *β* = 0, p-value = 0.98, N = 3,156 unrelated individuals, Figure S1h). Background CP-PGS was related to both total intracranial volume and growth through adolescence, indicating their influence on postnatal brain development (Figure S1g-h).

Together, these analyses suggest that language related variants in HAQERs may also increase the size of newborns, their brains, and subsequent birth complications. The relationship between HAQERs and birth complications provides a possible explanation as to why they have been under balancing selection, in contrast to the background CP-PGS which has undergone positive selection and appears to have more of an influence on postnatal neurodevelopment.

## 9 Evidence of convergent evolution in HAQERs for vocal learning

Next, we investigated whether homologous sequences to HAQERs in other species might influence their communication abilities. Previous work has classified > 200 mammalian species as either vocal learners or non-vocal learners[39]. Vocal learning species can acquire and modify their vocalizations through experience and auditory feedback, in contrast to species restricted to innate vocalizations. We focused our analysis on 170 non-primate species which were not used to derive HAQERs (121 non-vocal learning and 49 vocal learning species). For each species, we computed genome-wide “HAQER-like” and “HAR-like” sequence similarity scores by comparing regions aligning to the human reference genome using multiple sequence alignment data[40]. Strikingly, vocal learning species showed significantly higher “HAQER-like” sequence similarity scores compared to non-vocal learners, even after controlling for phylogenetic relatedness and “HAR-like” sequence similarity (phylogenetic regression beta = 3.92, p-value = 5.9 × 10^−6^, Figure 4f). Given that these vocal learning capabilities evolved independently from the human lineage, this finding reveals a fundamental property of HAQER-like sequences: they can reproducibly promote vocal learning across diverse evolutionary contexts, reaching their most sophisticated expression in humans. This supports an interpretation of the F1 core language factor as the human instantiation of a broadly reproducible “vocal learner” phenotype that has evolved independently in multiple mammalian lineages.

## 10 Discussion

In this study, we combine evolutionary genomics with observations in deeply phenotyped modern humans to understand how rapidly evolving genomic regions have contributed to and continue to shape the development of human language. The first major contribution of this work is in reconciling two major theories of the development of human language: the single-gene theory, popularized by early findings implicating *FOXP2* [1, 2, 41], and more recent work that espouses a highly distributed, polygenic model[13, 14, 15, 16, 17, 42]. In brief, we find and replicate evidence that HAQERs[5] – ~1,500 previously neutral regions that have gained regulatory potential in the human lineage – harbor alleles that have a strikingly disproportionate effect on language: a SNP in a HAQER has on average 112 times the impact on language that a SNP elsewhere in the genome has. While this result implicates regions of highly concentrated signal, these loci are distributed throughout the genome, thus supporting the polygenic view of human language evolution[13, 43, 44, 42]. Interestingly, we also found evidence that selective forces in the human lineage have guided HAQER sequences toward a significant preference for Forkhead box binding motifs (e.g., *FOXP2* ), and that the integrity of these motifs underlies individual differences in language ability in modern humans. This finding supports a key role for Forkhead box transcription factors in human language at both the species and individual level. Early reports focusing on rare coding variation in *FOXP2* struggled to find later generalization in more commonly observed individual differences in language[12, 11]. Our findings suggest that *FOXP2* and other Forkhead family members likely contribute to individual differences in language more consistently through polygenic variation in their downstream binding targets (e.g., in HAQERs in the current study) than through their own protein-coding variation.

Having established HAQERs as a key mechanism in language evolution, we next sought to understand how they manifest in measurable language abilities. This led us to examine which aspects of language most directly reflect the ancient genomic foundations we identified, versus more recent elaborations. The sentence repetition task presented an interesting case study in this regard. While human language researchers have debated the precise cognitive processes it measures - with evidence suggesting roles for verbal working memory, grammatical knowledge, phonological processing, and other linguistic systems[45, 46, 23, 24] - our cross-species analysis suggests it may tap into something more fundamental. Through our factor analysis of deeply and longitudinally phenotyped children (kindergarten, second, and fourth grades), we found that sentence repetition loads primarily on a core factor of language ability that remains stable across development even as other language skills change. While this task does engage complex linguistic knowledge in humans, our finding that HAQERs influence sentence repetition ability and show convergent evolution in vocal learning species suggests that the task’s core demand - the ability to perceive and accurately reproduce novel acoustic sequences through auditory feedback - reflects a fundamental capacity that emerged through regulatory changes in HAQERs during hominin evolution, and has independently evolved in other vocal learner species through similar genetic mechanisms.

This interpretation is strengthened by several converging lines of evidence. First, HAQERs show a striking specificity in their effects – they influence sentence repetition ability (F1) but not nonverbal IQ, mirroring how vocal learning capacity is dissociable from other cognitive abilities across species. Second, HAQERs’ influence on prenatal brain development, evidenced by their enrichment for variants affecting prenatal gene expression and their association with intracranial volume, suggests they help establish early neural substrates that support vocal learning ability. This is further supported by the pleiotropic effects we observe, where HAQERs that promote language ability also influence birth weight and are associated with birth complications, suggesting a fundamental role in prenatal development. Third, the enrichment of Forkhead box transcription factor binding sites in HAQERs provide a plausible mechanistic framework for known mechanisms of vocal learning, including *FOXP2*. Perhaps most compellingly, we find that homologous HAQER-like regions show convergent evolution specifically in vocal learning species across diverse mammalian lineages, suggesting they represent a common genetic substrate for the recurrent emergence of vocal learning ability.

The evolutionary trajectory of HAQER variants offers surprising insights into the emergence of human language. While cognitive variants outside HAQERs show clear positive selection over the past 20,000 years, HAQER variants have remained remarkably stable. Even more striking, we find that Neanderthals and Denisovans carried a higher proportion of language-promoting HAQER variants than modern humans, despite having lower polygenic scores for general cognition. This pattern suggests that the genetic foundations for complex language capabilities were established before the human-Neanderthal split (predating many previous language emergence estimates[47, 6, 7]) and have been maintained by balancing selection ever since. This apparent evolutionary paradox—the lack of positive selection on language-promoting HAQER variants—can be explained by our discovery of their pleiotropic effects on prenatal development. HAQERs that enhance language ability also influence birth weight and are associated with increased risk of birth complications, creating an evolutionary trade-off between core language ability and reproductive fitness, potentially including mechanisms linked to cephalopelvic disproportion. Importantly, our results align with observations linking prenatal development with language outcomes[48, 49]. These results suggest that the genetic architecture supporting language has been shaped not only by its adaptive benefits but also by developmental constraints. Modern medical interventions may now be relaxing these evolutionary constraints, potentially allowing for novel trajectories in the ongoing evolution of language-related genomic variation.

Our study has several important limitations. While we demonstrate clear associations between HAQER variation, Forkhead box binding motifs, and language ability, direct experimental validation of binding and its developmental consequences would strengthen our understanding of the causal mechanisms. In particular, the specific pathways through which HAQER variants influence prenatal development and birth outcomes warrant further investigation through functional studies. The evolutionary history of HAQERs presents another challenge. Although we have good coverage of recent human history through ancient DNA, our understanding of selection on HAQERs during earlier periods of hominin evolution remains limited by available samples. The oldest available genomic data from our genus is ~400,000 years old[50], leaving much of our early evolutionary history unresolved. Additionally, the relationship between HAQERs and reproductive fitness may have varied across different human populations and time periods in ways that are difficult to fully reconstruct.

These limitations point to several promising avenues for future research. Understanding how HAQER variants interact with other genomic regions could reveal additional insights into language evolution, while their association with birth complications suggests important clinical implications that warrant further investigation. Recent research has demonstrated how variants inherited from ancient human subpopulations can influence complex traits[51, 52, 53] - applying similar analyses to HAQERs could reveal how different human groups contributed to the mosaic of genetic variation that shapes modern language abilities. Finally, examining HAQER effects across diverse linguistic contexts could illuminate how these ancient adaptations interface with different language systems, though our findings linking HAQERs to vocal learning across species suggest their effects operate at a fundamental level that transcends specific languages.

This work bridges millions of years of human evolution with present-day individual differences, revealing how ancient genomic innovations established and continue to refine human language ability. The discovery that these same regions show convergent evolution across vocal learner species suggests we have identified a fundamental genetic mechanism for the emergence of complex communication - one that has been repeatedly utilized throughout evolutionary history and continues to shape human cognitive development today.

## 11 Methods

### 11.1 Human Subjects and IRB

All EpiSLI subjects in this study were minors who assented to participation under the University of Iowa IRB# 200511767. Secondary genetic analyses for EpiSLI subjects were approved and carried out under the University of Iowa IRB #201406727. SPARK is approved under WIRB #201703201. Approval for data collection and analysis of the SPARK Research Match study described here was conducted under the the University of Iowa IRB #201705739.

### 11.2 EpiSLI Cohort

The discovery cohort of this study - referred to as EpiSLI - was originally recruited as an epidemiological study of language impairments in Kindergarteners in the state of Iowa. A more detailed description of the recruitment scheme can be found in the initial [54], and subsequent [22, 55] publications. In brief, 7,218 kindergarteners, sampled to be representative of Iowa’s population were screened for language impairment with a rapid, 40-item subset [54] of the TOLD-2P [56]. Children who failed the TOLD-2P screener (i.e., have poor language ability) were over-sampled to capture a broad range of language ability in the primary cohort (n = 1,929 children). Children with autism spectrum disorder and/or intellectual disability were excluded. The entirety of the primary cohort received a more complete battery of language and cognitive assessments, while teachers and parents completed scholastic and behavioral questionnaires about the children 2nd, 4th, 8th, and 10th grades. Language and cognitive assessments included: TOLD-2P[56], WPPSI[57], a narrative story task[58], Woodcock Reading Mastery Tests-Revised[59], Word-Sound Deletion task[60], Random Animals-Colors task [60]. We selected a total of 390 children from the primary cohort for whole genome sequencing. This sample represents a broad spectrum of language abilities, these 390 children were chosen by sampling from the tails of the distribution of a composite language score inward until we reached our final sample size [54, 11].

Behavioral assessment scores came from T-scores for the summary scales of the Child Behavior Checklist (CBCL) developed by ASEBA[61]. CBCLs were completed at the 2nd grade timepoint of the study (241 of the 350 children used in this study had complete CBCL data for the analysis in Supplementary Table 2).

#### 11.2.1 Factor Analysis

An exploratory maximum-likelihood factor analysis was carried out on the language and cognitive assessment scores using the factanal function from the stats package in R [62], with the default “varimax” rotation. A number of factors from 1–10 were evaluated, with 7 - the largest number of factors with a nominal *χ*^2^ p-value less than 0.05 - chosen for subsequent analyses (Figure S1A). These seven factors accounted for an estimated 62.3% of the total variance in the original data.

#### 11.2.2 EpiSLI Cognitive measures

Core language assessment scores and IQ were normalized via the general scheme described in [54] to account for the over-sampling of individuals with low language ability. Core language scores were corrected for age as the residual of a linear regression model using lm() in R [62]. The age-corrected core language scores were then normalized with respect to the population using the wtd.mean() and wtd.var() functions from the Hmisc package [63] in R.

#### 11.2.3 Cognitive data Imputation

Most individuals for whom whole genome sequencing was generated had scores from assessments given in 2nd grade, while a minority had only 4th grade (41/390) or kindergarten (39/390) scores. Missing scores were imputed using chained random forests and predictive mean matching as implemented the missRanger R package [64] with 5,000 trees and pmm.k = 20.

### 11.3 EpiSLI Whole Genome Sequencing

#### 11.3.1 Sample Collection and whole genome sequencing

DNA was collected and extracted from either blood or saliva samples for 390 individuals (350 of which were used in the final analysis after quality control). DNA concentration of all sequenced samples was quantified with with Qubit 2.0 Fluorometer (Life Technologies Corporation). 390 DNA samples were sheared on a E220 Focused-ultrasonicator (Covaris) to an average size of 400 bp. Sequencing libraries were generated with a Kapa Hyper Prep kit (Kapa Biosystems) according to the manufacturer protocol. All samples had an average genome-wide coverage of at least 20X and were sequenced on a HiSeq4000 (Illumina) with 150-bp Paired End chemistry.

#### 11.3.2 Post-sequencing QC

All sequencing data was analysed with Fastqc (v0.11.8)[65], where no samples failed the run modules. After genome alignment (see below), some samples were found to have significantly better coverage than others, in excess of 40x. To help control for ascertainment bias in these samples, all samples with an average coverage > 35x (based on initially mapped BAM) were randomly down-sampled, leading to the distribution of genome-wide coverage and average insert-size outlined in Supplementary Table 17.

#### 11.3.3 Genome Alignment and Variant Calling

Reads were processed with bcbio (v1.1.6)[66] and mapped to hg19 via BWA-mem (v0.7.17)[67]. SNVs and InDels were then called with all samples in a pool with three variant callers: GATK (v4.1.2.0)[68], FreeBayes (v1.1.0.46)[69], and Platypus (v0.8.1.2)[70].

Variants from each caller were filtered according to caller-specific quality metrics. For GATK, variants needed to pass all VQSR tranche thresholds. For Platypus, variants were filtered based on the goodness of fit of genotype calls, excessive region-based haplotype scores, root-mean-square mapping quality, variant quality and its ratio with read depth, low complexity sequence context, allele bias, region-based read quality, neighboring homopolymers, and strand bias. For FreeBayes, variants were filtered based on a combination of allele frequency, read depth, and overall quality. The thresholds used for filtering Platypus and FreeBayes calls were the default set by bcbio. An ensemble of all three callers was generated and used in all subsequent analyses in order to achieve improved specificity in the detection of rare variants. All variants in the ensemble callset were called by GATK as well as either Platypus OR FreeBayes. The majority of variants ( 87%; 25,775,508) were called by all three callers, while a minority were called by GATK and only one other caller ( 13%; 3,937,146). All variants were filtered to have a QUAL and individual quality score ≥ 20. Qualified researchers can access the individual level genotypes from dbGaP at (study accession = phs002255.v1.p1): https://www.ncbi.nlm.nih.gov/projects/gap/cgi-bin/study.cgi?study_id=phs002255.v1.p1

#### 11.3.4 Final sample QC

Starting from the initial sample size of 390, a total of 14 samples were flagged for removal from all association analyses because of population stratification. Specifically, these samples did not cluster with 1,000 Genomes Europeans[71], or were more than 3 standard deviations away from the rest of the EpiSLI cohort based on the top 10 multidimensional scaling components calculated from SNPs found at or above a 0.05 minor allele frequency. An additional 26 samples were dropped due to relatedness or limited phenotypic data, leaving a final sample size of 350 unrelated European individuals with complete data for all genomic analyses.

#### 11.3.5 Variant Annotations

Variants were annotated with the Ensembl Variant Effect Predictor tool (VEP v109) [72]. Reference population allele frequencies came from 1000 Genomes Phase 3 samples[71] and GnomAD[73], rsIDs of variants were dbSNP [74] (v151), these additional annotations were added using VCFanno [75] (v20190119).

### 11.4 Polygenic Scores (PGS)

#### 11.4.1 Genotypes for PGS

To derive a set of SNPs suitable for PGS analysis, we merged our dataset with 1000 Genomes Europeans to use as a reference sample. Based on widely used recommendations[76], we extracted SNPs with a minor allele frequency ≥ 1%, Hardy-Weinberg equilibrium p-value > 1 × 10^−6^, and a missingness rate < 2% in both samples (leaving 7,719,665 SNPs total for PGS calculation).

#### 11.4.2 PGS Calculation

LDpred2 was used to calculate a genome-wide PGS for all traits with the infinitesimal model using the provided UK Biobank LD reference panel and HapMap3+ variant set [77]. PGS were calculated using GWAS summary statistics for psychiatric traits: ADHD [78], addiction[79], alcohol dependency [80], Autism [81], Anorexia [82], bipolar disorder [83], depression [84], insomnia [85], neurodevelopmental conditions [86], PTSD [87], and schizophrenia [88]. GWAS summary statistics for cognitive traits included: cognitive performance[89], educational attainment [90], executive functioning [91], and the “g Factor” [92]. Additional PGS were calculated for the following behavioral and socioeconomic status related traits: childhood aggression[93], antisocial behavior [94], empathy [95], the BIG5 personality traits [96], income [97], and the Townsend Deprivation Index (a measure of material deprivation) [98]. PGS were also calculated for brain structural[99, 100] and functional connectivity phenotypes [101]. Finally, we computed PGS for miscellaneous traits: left-handedness [98], height [102], childhood trauma [103], and vocal pitch [104]. It is important to note we did not compute PGS in EpiSLI for reading based traits, because our sample was part of the discovery cohort of the largest reading related GWAS to date [13]. Associations for all of these PGS with our language factors can be found in Supplementary Table 3.

To account for population stratification, we corrected PGS for the first 5 genetic principal components. PGS were normed to the 1000 Genomes Europeans reference sample.

#### 11.4.3 ES-PGS Calculation

Here we describe ES-PGS, a novel method to estimate the point in evolutionary history that genetic code key to a phenotype developed. This technique leverages two key pieces of information; (1) evolutionary genomic annotations and (2) stratified PGS, providing a PGS for a trait of interest across each provided annotation in the genome. The annotation restricted PGS (ES-PGS) is used to represent key evolutionary events and a “background PGS” is calculated to control for the rest of the genome. The ES-PGS and the background PGS are jointly modeled to determine whether the evolutionary annotation is predictive of the phenotype and independent of the rest of the genome (as measured by the background PGS). These results were used to answer the question: “how much does this specific part of the genome contribute to language ability, over and above what is expected from the rest of the genome?”.

Briefly, we use the ANOVA test of a reduced and full model to determine whether the addition of a new model term significantly improves the model fit. In the case of ES-PGS, the reduced model is:

1 $$y=\beta_{0}+\beta_{1}{\text{PGS}}_{\text{background}}+\epsilon$$

where y is the phenotype of interest, in our case the language factor score (e.g., F1, F2, or F3), ${\text{PGS}}_{\text{background}}$ is the polygenic score calculated using all independent SNPs except those in the evolutionary annotation of interest, and ϵ is the error term. The full model includes an additional term for the ES-PGS:

2 $$y=\beta_{0}+\beta_{1}{\text{PGS}}_{\text{background}}+\beta_{2}{\text{PGS}}_{\text{annotation}}+\epsilon$$

where ${\text{PGS}}_{\text{annotation}}$ is the polygenic score calculated using only independent SNPs within the specific evolutionary annotation. By comparing these models, we can determine whether the addition of the annotation-specific PGS significantly improves the model’s fit, indicating a unique contribution of that evolutionary annotation to the trait beyond what is explained by the background PGS. This approach allows us to systematically test the contributions of a range of evolutionary periods to our language ability phenotypes, providing insights into the evolutionary history of genetic variants associated with language development.

PRSet [28] was used to calculate stratified PGS using human genome annotations in BED formatted files, computing a clumping and thresholding based ES-PGS for each annotation and a background PGS for the rest of the genome. Background regions for each annotation were identified using the “complement” function from bedtools (v2.26.0)[105]. As recommended by the authors of PRSet, we used a p-value threshold of 1 and the 1000 Genomes Europeans as the LD reference [71]. We corrected for population stratification using the same steps as described in the genome-wide PGS analysis.

We focused our analysis on genome annotations related to primate and human evolution, annotations included: primate ultra conserved regions (primate UCEs) [21], primate lineage accelerated regions [30], human-chimp divergent genes (differentially methylated genes between humans and chimp)[31], Human Ancestor Quickly Evolved Regions (HAQERs)[5], Human Accelerated Regions (HARs)[32], Neanderthal Selective Sweep loci (the 5% of the human genome most depleted for Neanderthal derived variants)[33], and recent human selection pressures (highest scoring 5% of SNPs based on absolute value of Singleton Density Scores)[35]. All annotations were converted to BED format and lifted over to match genome builds when necessary, using the UCSC liftOver tool[106].

#### 11.4.4 ES-PGS replication in SPARK

To replicate our ES-PGS results in a separate sample we used the SPARK cohort, a large genetic study of individuals with autism and their family members [18]. For the replication we utilized imputed SNP array data that we have previously described[107] to compute an ES-PGS using the same workflow as in our EpiSLI sample. One phenotype, the “core language ability” factor, came from a previously described research match where we had > 1,000 adults with autism or parents of children with autism complete an online language battery[38]. All other phenotypes came from the SPARK v13 phenotype release. All analyses included age and sex as covariates.

### 11. 5 SPARK rare ancestral reversion analysis

To explore the effects of rare genetic variation in evolutionary significant regions on language ability, we used the whole genome sequencing data from the previously described SPARK cohort (max N = 11,545)[18]. Briefly, we merged the whole genome sequencing data provided by SPARK (WGS batches 1–4) using bcftools, then used the same processing pipeline as we did for the EpiSLI cohort: filtered to variants with a QUAL and individual quality score ≥ 20, and annotated the variants with VEP (v109)[72]. To identify rare variants we then filtered variants in both datasets to have a maximal reference population allele frequency < 1% and an allele frequency < 1% in SPARK. Ancestral alleles were identified using those provided in the original HAQER manuscript[5]. We identified all reversion variants within 10Kb of HAQERs, HARs, or random non-coding (RAND) sequence and counted the number of rare ancestral reversions each sample had in each of these elements. We removed outlier samples who had > 2.5 median absolute deviations away from the median value for either HAQER, HAR, or random reversions (N = 1,781 outliers), but had consistent phenotypic associations even when including these outliers in the analysis. We then used these reversion counts for association with speech and language phenotypes in SPARK.

### 11.6 Transcription Factor Analysis

#### 11.6.1 Variant selection and annotation

We analyzed transcription factor binding sites in three distinct genomic contexts: Human Accelerated Regions (HARs), Human-Accelerated Quickly Evolved Regions (HAQERs), and matched random genomic regions (RAND, all taken from[5]). Position weight matrices (PWMs) for 633 human transcription factors were obtained from the JASPAR2020 database[108], with pseudocounts adjusted according to base frequency distributions.

Variants were filtered using strict quality control criteria as described above. For this analysis, we retained only biallelic single nucleotide variants (SNVs) located within feature boundaries that exhibited minor allele frequencies below 1%. Complete great ape allele information was required for each variant, including data from Neanderthals and Denisovans, Chimpanzee, Bonobo, Gorilla, and Orangutan genomes [109, 110, 111, 112, 5]. The final dataset comprised genotype information from 15,746 rare variant sites across 350 individuals, with corresponding variant annotations.

#### 11.6.2 Reversion Status Determination

To characterize the evolutionary trajectory of variants, we developed a machine learning approach to impute reversion status where direct determination was not available. We implemented an elastic net regression model (*α* = 0.9) using the glmnet package in R[113], incorporating great ape allele states as predictors of reversion status as given in[5]. To address class imbalance, we applied weights to ensure equal representation across sequence context types (HAQER, HAR, random). Reversion status was assigned using a probability threshold of 0.8, with known states preserved for training data.

#### 11.6.3 Sequence and Motif Analysis

For each variant, we extracted 51-base pair genomic windows centered on the variant position from the human reference genome (hg19). Alternative sequences were generated by substituting variant alleles into the reference background. We then calculated maximal motif scores for both reference and alternative sequences across all JASPAR2020 human transcription factor motifs. Scores were computed on both forward and reverse complement strands, with the maximum score retained for reference and alternate alleles of each variant-motif pair.

#### 11.6.4 Language Association Analysis

Core language ability was assessed using the F1 measure as described above in the factor analysis. We computed burden scores for each transcription factor motif by combining variant effects weighted by genotype status of reversion sites. Linear regression models were employed to estimate the associations between individual context-specific (aggregate) motif scores and language ability.

#### 11.6.5 Transcription Factor Motif Score Selection Analysis

For each transcription factor, the (Z-scaled) difference in reference and alternate allele motif scores was modeled as a linear function of variant sequence context (HAQER, HAR, or random) and a binary reversion status indicator. Separate reversion effects were estimated for HAR, HAQER, and random region variants as a sequence context by reversion interaction term. Estimated beta coefficients from these terms, as well as their standard errors, were extracted for use in downstream analyses.

#### 11.6.6 Joint Selection-Language Enrichment Analysis

We used York regression analysis[114] to examine relationships between selective pressure for motif integrity and language-related effects. This approach accounts for uncertainty in both variables (i.e., language association betas and selection effect betas). Prior to the analysis, the sign of the selection betas were flipped such that positive values indicate human-divergent selection for increased motif scores (i.e., reversions to the human-chimp ancestral allele tend to decrease motif scores). York regression betas, their standard errors, a Chi-squared goodness of fit statistic and its p-value were extracted to interpret the significance of the overall relationship between TF motif integrity (i.e., motif score) and motif score effect on individual differences in language ability, for each sequence context. Individual TF motifs of interest are those with nominal significance (p < 0.05) for both selection for motif integrity and for positive association of aggregate motif integrity with higher F1 core language scores. Transcription factors were classified into families using InterPro annotations[115] of representative families found to be significantly associated with a reversion effect in either direction (using www.string-db.org)[116]. To identify transcription factor families showing convergent patterns of selection and language association, we performed 2×2 Fisher’s exact tests comparing the proportion of motifs with concordant effects (positive selection AND positive language association vs. all other combinations) across families. Odds ratios with 95% confidence intervals and p-values were computed for each TF family.

### 11.7 Ancient DNA

#### 11.7.1 Neanderthal and Denisovan DNA data and ES-PGS

VCFs with genotypes for the three high coverage Neanderthals (Altai[109], Chagyrskaya[110], and Vindija[111]) and one Denisovan[112] produced from high-coverage while genome sequencing were downloaded from the Max Planck Institute for Evolutionary Anthropology website. The individual genomes were merged using bcftools. We then subset the VCFs to SNPs used in our EpiSLI dataset merged with 1000 Genomes Europeans for analysis (N = 7,593,137 overlapping loci), limiting spurious genotypes and allowing us to more directly compare the samples. We then computed ES-PGS in the merged archaic human, 1000 Genomes European, and EpiSLI dateset using the same SNPs as we did in the primary EpiSLI HAQER ES-PGS analysis. This left us with 1,335 SNPs called in both the archaic samples and modern samples out of the 1,350 total HAQER independent SNPs used in the EpiSLI discovery sample. We imputed missing genotypes in all samples using the mean genotype value for the missing SNP so they would have minimal impact on the final ES-PGS. Given the difficulty of accounting for population stratification with the archaic humans, we used the raw polygenic score presented in Figures 5b-c to provide more conservative estimates of archaic human cognitive abilities. Notably, when we do account for the genetic PCs more traditionally, based on 5 first PCs computed using the 1000 Genomes Europeans, the archaic human polygenic scores become even more extreme (higher HAQER CP-PGS and lower background CP-PGS, Supplementary Table 12). Additionally, to ensure this result was not simply an artifact of some systematic missingness from these ancient sample we computed HAQER CP-PGS using only SNPs called in all 4 of the archaic humans and came to the same conclusion that archaic human species appear carry more HAQER CP-PGS than modern Europeans (N = 558 SNPs, Supplementary Table 12).

#### 11.7.2 Ancient homo sapiens data

DNA data and sample age information for ancient homo sapiens came from the Allen Ancient DNA Resource (AADR) version 54. We downloaded the publicly available EIGENSTRAT formatted files and converted them to PLINK format using the EIGENSOFT tool. We then merged the ancient genomes with our EpiSLI and 1000 Genomes Europeans dataset to ensure we were using comparable SNPs for our ES-PGS selection analysis as we did in our discovery sample. We identified ancient west Eurasians using the same criteria as a recent large-scale selection analysis[117]. Briefly, we filtered to samples found between longitude 25W and 60E and latitude 35N to 80N, samples passing quality control with an assessment labeled as “PASS”, and sample ages > 0 but < 20,000 years old.

We then computed ES-PGS for CP in HAQERs using the same methodology as we did in the EpiSLI, SPARK, and ABCD samples for use in our polygenic selection analysis. Given the challenges of accounting for population structure in ancient DNA, we opted to use a LMM based approach instead of a traditional PC based approach as recommended by Akbari et al., 2024[117]. With the ancient west Eurasian subsample, we then identified independent SNPs using 1000 Genomes as the LD reference with PLINK’s “–indep-pairwise” function (window size = 1000bp, step size = 1bp, r^2^ threshold = 0.05, MAF ≥ 5%)[118]. Next, we identified samples and SNPs with low missingness for GRM calculation (samples missing < 50% of independent SNPs, and SNPs missing in < 10% of those samples). Finally, we computed the genetic relatedness matrix (GRM) with GCTA[119] with the QC passing samples and SNPs and removed duplicate/twin samples for subsequent analysis (GCTA “grm-cutoff” 0.9).

We then used the 3,244 QC passing samples and the GRM based on the 12,146 QC passing SNPs for the ES-PGS analysis. We implemented the LMM based polygenic selection analysis with the gaston[120] R package (lmm.aireml function), allowing us to account for the GRM which reflects population structure and relatedness of the sample. We used log10(sample age) as the outcome variable and HAQER CP-PGS and background CP-PGS as the independent variables (similar to the ES-PGS analysis we used in our other samples).

### 11.8 Detecting balancing selection

To detect signatures of balancing selection, we analyzed the WGS data we generated in EpiSLI. First, we subset to regions of interest (HAQERs, HARs, and RAND sequences). Then we computed a site frequency spectrum (SFS) for each sequence class, calculating the proportion of variants in each minor allele frequency bin. We compared HAQERs SFS to the SFS of both HARs and RAND sequences, to identify whether HAQERs had a relative enrichment of intermediate frequency variants which can indicate balancing selection (or ongoing selection).

Next, to more formally test our balancing selection hypothesis we identified common independent SNPs in these regions using PLINK[118], using the “–indep-pairwise” function (window size = 200bp, step size = 50bp, r^2^ threshold = 0.5, MAF ≥ 5%)[118]. This identified common independent SNPs in HAQERs (2,641 SNPs), HARs (1,411 SNPs), RAND sequences (1,670 SNPs). We then computed individual level F-statistics for each class of variation using the “–het” function in PLINK[118], which derives F-statistics based on the expected number of homozygotes and observed homozygotes (with more negative values indicating there are more heterozygotes than expected). We compared F-statistics between classes using t-tests to determine if there was excess heterozygosity in HAQERs, a signature of balancing selection. Additionally, we correlated the sample level F-statistics with core language (F1) and nonverbal IQ (F3) scores to determine whether the excess heterozygosity is beneficial to language.

### 11.9 ES-PGS analysis in ABCD

To explore the effects of HAQER CP-PGS in prenatal development we analyzed the ABCD cohort, a large longitudinal study of adolescent development with genetic, brain imaging, and developmental phenotypes [121]. All phenotypes used in ABCD came from the v4.0 data release. Similar to the SPARK replication analysis, we utilized imputed SNP array data that we have previously described[107] to compute ES-PGS using the same workflow as in our EpiSLI and SPARK samples. We computed genetic relatedness using GCTA[119] in the merged ABCD and SPARK dataset. Using the relatedness matrix, we identified unrelated individuals for ES-PGS analysis with brain imaging phenotypes (genetic relatedness < 0.05). We used the most recent intracranial volume value provided by ABCD for association with our CP ES-PGS. Covariates for the intracranial volume analysis included: age, sex, height, and weight. For the postnatal brain growth phenotype, we identified each individual’s initial structural brain MRI as well as their most recent and computed difference scores between the intracranial volumes, limiting our analysis unrelated individuals who had multiple scans and a brain growth score > 0. We adjusted the brain growth score for total volume, age, sex, and difference in age between MRI scans.

For our within-family ES-PGS analysis, we used the relatedness matrix to identify full siblings in the dataset (0.675 < relatedness > 0.375) who were not dizygotic twins (different ages). Using the dataset of full siblings (N > 500 sibling pairs), we computed difference scores for birth related phenotypes (birth weight and birth via c-section) as well as for CP ES-PGS (HAQERs and background). In the sibling birth weight difference ES-PGS analysis, we included difference in age, sex, and gestation duration as covariates.

### 11.10 HAQER enrichment of scQTLs

To determine if HAQERs, HARs, and RAND sequences significantly influence gene expression in prenatal and postnatal brains, we leveraged scQTLs from two studies. (1) a study of stem cell derived neurons, meant to mimic early prenatal cells in the midbrain[122] and (2) a study of nearly 400 postmortem adult brains from psychENCODE2[123]. For scQTL study (1), we defined scQTLs as all variants with a p-value < 5 × 10^−4^, for scQTL study (2) we utilized all scQTLs defined as significant by the authors. To determine enrichment for scQTLs in our regions of interest, we used the “intervalOverlap” function from gonomics[124]. This allowed us to compute expected versus actual overlaps (based on size of the human genome and the provided annotations) providing an enrichment p-value for each region and scQTL cell-type combination.

### 11.11 Convergent evolution of vocal learning analysis

To test for evidence of convergent evolution of “HAQER-like” sequences in vocal learning species, we utilized a large dataset of > 400 species with whole genome data aligned to the human reference genome (hg38)[40]. We parsed the alignments with Biopython[125] to subset to regions of interest. For each species and sequence type (HAQERs or HARs), we computed a sequence similarity (using the number of bases matching the human reference genome in these regions). We then used the “HAQER-like” sequence similarity to predict vocal learning status in 170 non-primate species that have been previously described[39]. To determine statistical significance and account for species relatedness we used phylogenetic logistic regression[126] with the phylolm package[127] in R. Additionally, we included “HAR-like” sequence similarity as a covariate to demonstrate the effect was specific to “HAQER-like” sequences.

### 11.12 Statistical analysis

All statistical analysis was done in R (version 4.3.1)[62].

### 11.13 Approach to multiple testing correction and triangulating evidence

To address multiple testing concerns, we employed False Discovery Rate (FDR) correction for analyses testing more than 20 hypotheses, allowing us to limit type 1 errors (false positives). This was applied to the EpiSLI CBCL mental health score analysis and genome-wide PGS analysis where we conducted many statistical tests (Supplementary Tables 2–3).

Rather than relying solely on stringent p-value thresholds, we adopted a comprehensive approach to validate our key findings through multiple lines of evidence. This strategy was particularly important given that some of our individual statistical tests showed modest statistical significance (0.01 < p-values < 0.05). We established confidence in our results through: (1) replication across independent cohorts (e.g., EpiSLI and SPARK); (2) identifying convergent evidence across different analytical approaches (e.g., common variant, rare variant, and transcription factor binding analyses); (3) demonstration of specificity, showing HAQERs’ associations with language but not nonverbal IQ; (4) evolutionary support from ancient DNA and cross-species analyses; and (5) mechanistic support through analysis of transcription factor binding and enrichment of variants influencing prenatal gene regulation. This multi-faceted approach allowed us to distinguish robust biological signals from statistical noise, even when some individual analyses showed moderate statistical significance. Our findings’ consistency across diverse data types, species, and analytical methods provides stronger evidence than would be achieved through any single statistical test, regardless of its p-value.

## Supplementary Material

Supplement 1

## Figures and Tables

**Figure 1:**
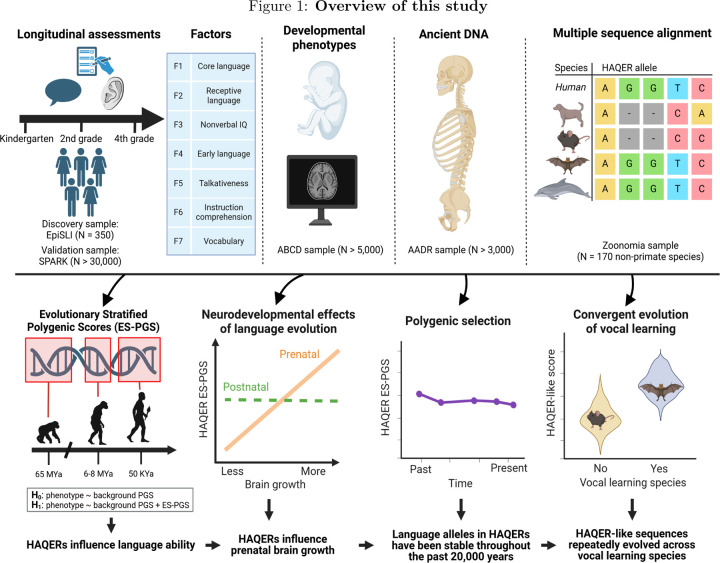
Overview of this study

## Data Availability

The EpiSLI whole genome sequencing data described here is available to qualified researches via dbGaP (study accession = phs002255.v1.p1): https://www.ncbi.nlm.nih.gov/projects/gap/cgi-bin/study.cgi?study_id=phs002255.v1.p1 SPARK genetic and phenotype data is available to qualified researchers at SFARI base: https://base.sfari.org/ ABCD is available to qualified researchers at: https://nda.nih.gov/abcd/request-access 1000 Genomes Phase 3 data is available at: https://www.internationalgenome.org/data/ Allen Ancient DNA Resource: https://reich.hms.harvard.edu/allen-ancient-dna-resource-aadr-downloadab Neanderthal and Denisovan genomes: https://www.eva.mpg.de/genetics/genome-projects/ Cross-species sequence alignment data: https://hgdownload.soe.ucsc.edu/goldenPath/hg38/cactus447way/
