# Ancient regulatory evolution shapes individual language abilities in present-day humans

**DOI:** 10.1101/2025.03.07.641231

**Published:** 2026-02-13

**Authors:** Lucas G. Casten, Tanner Koomar, Taylor R. Thomas, Jin-Young Koh, Dabney Hofammann, Savantha Thenuwara, Allison Momany, Marlea O’Brien, Jeff C. Murray, J. Bruce Tomblin, Jacob J. Michaelson

**Affiliations:** 1 Department of Psychiatry, University of Iowa, Iowa City, 52242, USA.; 2 Center for Genomic Medicine, Massachusetts General Hospital, Boston, 02114, USA.; 3 Department of Otorhinolaryngology, University of Maryland, College Park, 20742, USA.; 4 Carver College of Medicine, University of Iowa, Iowa City, 52242, USA.; 5 Stead Family Department of Pediatrics, University of Iowa, Iowa City, 52242, USA.; 6 Department of Communication Science and Disorders, University of Iowa, Iowa City, 52242, USA.

## Abstract

Language is a defining feature of our species, yet the genomic changes enabling it remain poorly understood. Despite decades of work since *FOXP2*’s discovery, we still lack a clear picture of which regions shaped language evolution and how variation contributes to present-day phenotypic differences. Using a novel evolutionary stratified polygenic score approach in nearly 40,000 individuals, we find that Human Ancestor Quickly Evolved Regions (HAQERs) are specifically associated with language but not general cognition. HAQERs evolved before the human–Neanderthal split, giving hominins increased binding of Forkhead and Homeobox transcription factors, and show balancing selection across the past 20,000 years. Remarkably, language variants in HAQERs appear more prevalent in Neanderthals and have convergently evolved across vocal-learning mammals. Our results reveal how ancient innovations continue shaping human language.

Human language is one of our species’ most remarkable cognitive innovations, yet the genetic mechanisms underlying this ability remain elusive. While the human genome differs by only 1–5% from our closest primate relatives (1–3), these modest genetic changes enabled the evolution of our species’ unique capacity for complex language. Understanding how these relatively small genomic differences produced profound cognitive differences represents a central challenge in evolutionary genetics, with implications for language disorders, human cognitive diversity, and the origins of human-specific traits (4).

The discovery that mutations in *FOXP2* cause speech and language disorders provided the first clear example of a single gene with significant effects on language, reinforcing early expectations for simple genetic architectures (5, 6). However, *FOXP2*’s contribution to typical variation in language ability proved limited, with subsequent studies failing to find associations between common *FOXP2* variants and individual differences in language skills (7, 8). This limitation shifted research toward polygenic models emphasizing a large number of regulatory elements scattered throughout the genome that collectively influence language development. Genome-wide association studies have since identified numerous loci contributing to reading abilities, stuttering, rhythm, and vocabulary development, supporting a highly polygenic architecture (9–14). Cross-species studies have revealed that language-related traits (vocal learning and rhythm) show convergent evolution across mammalian lineages, with distributed regulatory networks rather than single genes controlling complex vocal behaviors (15–20). However, this polygenic model has left critical evolutionary questions unanswered: how did language-relevant regulatory elements chnage during human evolution, when did humans acquire language-promoting functions, and how do ancient evolutionary changes translate into present-day individual differences in language abilities?

To address these questions, we analyzed 65 million years of primate evolutionary history to trace the origins of language-relevant genetic variation. As part of our approach, we developed an evolutionary stratified polygenic score (ES-PGS) method that partitions genetic effects based on the evolutionary origins of their sequence context. We applied this approach across nearly 40,000 individuals with detailed language phenotyping, combined with molecular analysis, ancient DNA analysis, and cross-species genomic comparisons. This multi-modal framework enabled us to directly connect ancient genomic innovations with modern individual differences in language ability, identify neurobiological mechanisms supporting language evolution, and revealed how evolutionary trade-offs have shaped human cognitive variation.

## Results

### Dimensions of language ability

**Figure 2:**
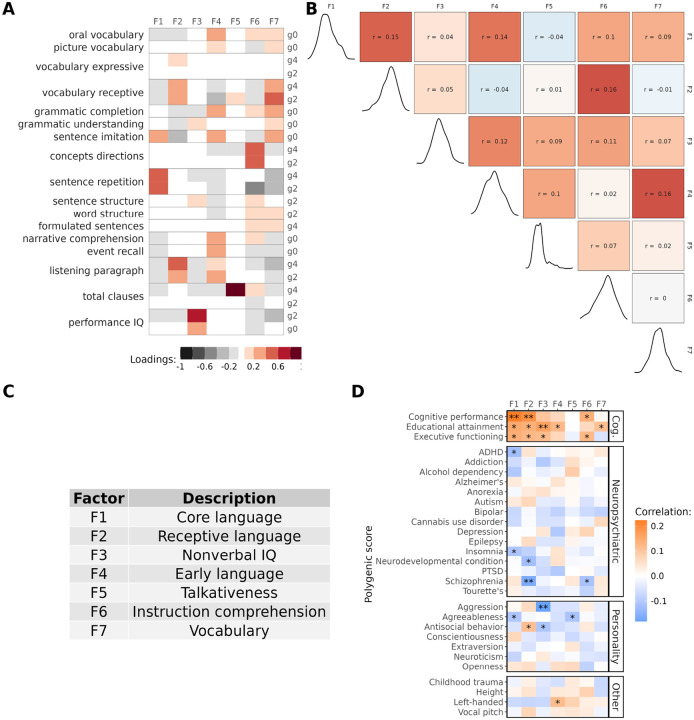
Factor loadings and genetic associations **A** Loadings of cognitive and language assessments onto the seven language factors. g0 = Kindergarten (age 5–6), g2 = 2nd grade (age 7–8), g4 = 4th grade (age 9–10). **B** Pearson correlations for language factors (upper triangle) and distribution of each factor (diagonal). **C** Interpretations of the language factors based on their loadings. **D** Pearson correlations for each factor with genome-wide PGS. ** indicates FDR adjusted p-value < 0.05 and * indicates unadjusted p-value < 0.05.

To quantify dimensions of developmental language abilities, we analyzed 17 longitudinal cognitive and language assessments administered from kindergarten through 4th grade for 350 children sampled from a community-based cohort (21), which we refer to as the ”EpiSLI” cohort. This analysis revealed seven factors representing distinct aspects of language ability (Figure 2A). The first factor (F1), primarily driven by sentence repetition scores, represents ”core language” ability. Sentence repetition strongly indicates overall language capacity, making F1 a key measure of general language competence (22, 23). The second factor (F2) relates to receptive vocabulary and listening comprehension, covering broad receptive language skills. The third factor (F3) specifically reflects nonverbal IQ, aligning with performance IQ at both kindergarten and 2nd grade. Factor F4 captures pre-literacy language skills, incorporating all kindergarten scores except performance IQ. Its slight correlation to F1 and F2 (*r* = 0.13 and 0.12), but not F3, suggests specificity to language (Figure 2B). Factor F5, which we call ”talkativeness,” mainly reflects the number of clauses produced in a narrative task. Factor F6, based on a comprehension of concepts and directions assessment, indexes mastery of directive language (i.e., task-based instructions). Factor F7 spans a variety of assessments, with specific loading on vocabulary and grammar-related tasks, suggesting a broad, crystallized knowledge of language.

Most of our preliminary investigation of these factors suggested that Factors 1, 2, and 3 carried the most genetic association signal (Figure 2D, Supplementary Table 2). We also find pervasive associations with F1-F3 and measures of mental health in our sample (N = 241, Figure S1, Supplementary Table 1).

### Evolutionary Stratified Polygenic Score Analysis

To investigate the genetic origins of language ability, we developed an evolutionary stratified polygenic score (ES-PGS) approach that systematically examines how genetic variants from different evolutionary periods contribute to traits. ES-PGS builds on the conceptual framework of partitioned heritability and pathway-based polygenic score methods, which have successfully partitioned genetic effects across functional genomic regions (24–27). However, evolutionary questions about when and how language-relevant functions emerged during human evolution require partitioning based on phylogenetic age rather than functional annotations. ES-PGS addresses this need by partitioning polygenic scores based on evolutionary origin, testing whether incorporating specific evolutionary periods significantly improves phenotypic predictions beyond what is explained by the rest of the genome and biologically matched random control regions (matched for chromosome, size, GC content, repeat content, distance to nearest gene, number of genes nearby, and overlap with promoter or coding regions). By leveraging individual-level data rather than summary statistics, ES-PGS enables direct association testing in deeply phenotyped cohorts, which is particularly valuable for specialized studies like ours with extensive language assessments.

### Human-specific genomic regions predict individual differences in language ability

We applied ES-PGS using the cognitive performance polygenic score (CP-PGS) (28) to trace the evolutionary origins of language abilities. This polygenic score captures broad dimensions of cognitive function, enabling valid comparisons across multiple cognitive domains (including language and nonverbal IQ). We first confirmed that the CP-PGS showed the expected associations in our EpiSLI sample, the genome-wide CP-PGS showed significant associations with both core language (F1, *r* = 0.22, FDR adjusted p-value = 0.001) and receptive language ability (F2, *r* = 0.19, FDR adjusted p-value = 0.01), (Figure 2D). Finally, we partitioned CP-PGS across five evolutionary annotations spanning approximately 65 million years of primate and human evolution, ranging from ancient primate-conserved regions to sequences differentiating modern humans from Neanderthals, to systematically trace which genomic regions and evolutionary periods contributed to different aspects of human cognition (29–33).

**Figure 3:**
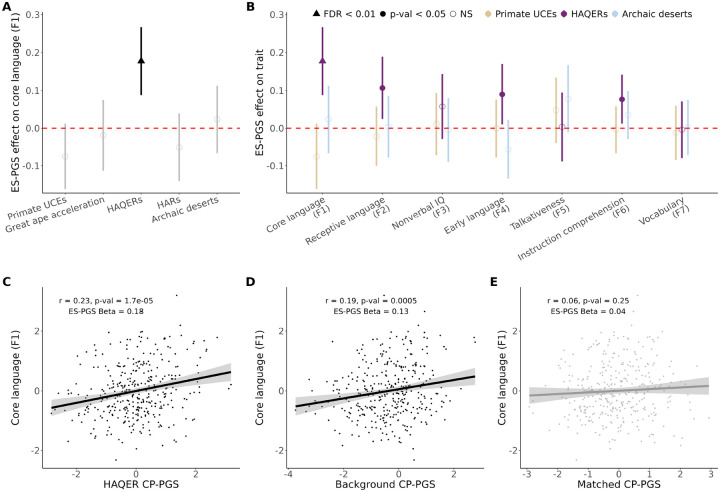
HAQERs are associated with language ability and not nonverbal IQ **A** Comparison of evolutionary events on core language ability in EpiSLI (N = 350). Points represent the *β* provided from the ES-PGS models for each evolutionary annotation, while the ranges represent the 95% confidence interval. Solid points indicate p-value < 0.05. **B** Comparison of 3 evolutionary (oldest = Primate UCEs, middle = HAQERs, and youngest = archaic deserts) events on the 7 factor scores in EpiSLI (N = 350). Points represent the *β* provided from the ES-PGS models for each evolutionary annotation, while the ranges represent the 95% confidence interval. Solid points indicate p-value < 0.05. **C** Scatterplot of HAQER CP-PGS with core language scores (F1) in the EpiSLI sample. **D** Scatterplot of background CP-PGS with core language scores (F1) in the EpiSLI sample. **E** Scatterplot of biologically matched control regions CP-PGS with core language scores (F1) in the EpiSLI sample (matched to HAQERs).

Human Ancestor Quickly Evolved Regions (HAQERs) emerged as the most compelling finding from this analysis. Despite comprising less than 0.1% of the human genome, HAQERs showed associations with four of the seven factors (F1, F2, F4, and F6, Figure 3B, Supplementary Table 3). HAQER CP-PGS demonstrated the strongest association with core language ability (*r* = 0.23, ES-PGS model *β* = 0.18, p-value = 1.2 × 10^−4^, FDR adjusted p-value = 0.004, Figures 3A–C) while showing no association with nonverbal IQ (F3, ES-PGS model *β* = 0.06, p-value = 0.19, FDR adjusted p-value = 0.61). HAQERs are largely non-coding sequences that began rapidly evolving after the human-chimpanzee split (approximately 6 million years ago) but before human-Neanderthal divergence (approximately 600,000 years ago), acquiring novel regulatory functions in the human lineage (31, 34).

The predictive power of HAQERs is striking. While the background and matched CP-PGS together utilized approximately 300,000 independent SNPs and explained 3.7% of variance in core language ability, adding HAQER CP-PGS (comprising only 1,763 independent SNPs) increased explained variance to 7.7%. This indicates that an average HAQER SNP carries 188 times more predictive power for language than SNPs elsewhere in the genome, with HAQERs alone explaining slightly more variance of core language scores in the EpiSLI cohort (*r*^2^ gain of 4%) than the remaining >99.9% of the human genome (*r*^2^ of 3.7%).

In contrast, Human Accelerated Regions (HARs), which are deeply conserved regulatory elements that acquired human-specific changes (32, 35), showed no comparable signal using ES-PGS (F1 *β* = −0.05, p-value = 0.27, FDR adjusted p-value = 0.7, Figure S2). This distinction suggests that human language ability emerged through novel regulatory innovations (HAQERs) rather than modifications to existing functional elements (HARs). The specific association between HAQERs and language factors but not nonverbal IQ reveals a distinct evolutionary trajectory for verbal abilities compared to general cognition. Supporting this distinction, nonverbal IQ (F3) was most strongly associated with genomic regions that underwent rapid changes across all great apes (30) (ES-PGS *β* = 0.13, p-value = 0.004, FDR adjusted p-value = 0.07, Figure S2).

### HAQERs influence language ability across multiple cohorts and variant types

**Figure 4:**
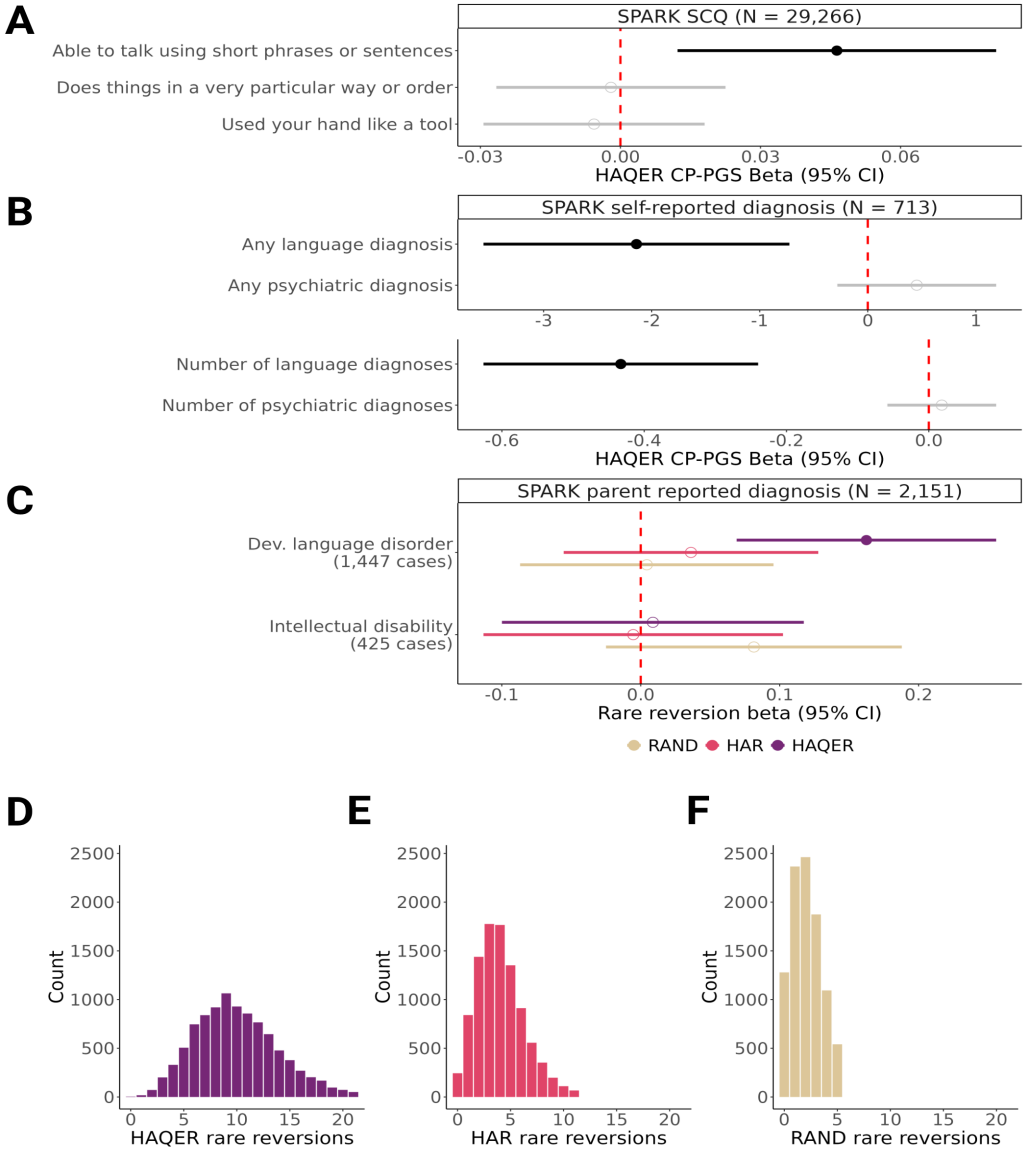
Large-scale validation of HAQERs association with language ability **A** Points represent the *β* provided from the ES-PGS models for the HAQER CP-PGS on Social Communication Questionnaire (SCQ) items in SPARK, while the ranges represent the 95% confidence interval. Solid points indicate p-value < 0.05. **B** Points represent the *β* provided from the ES-PGS models for the HAQER CP-PGS on self-reported language and psychiatric diagnosis in SPARK, while the ranges represent the 95% confidence interval. Solid points indicate p-value < 0.05. **C** Points represent the *β* provided from the regression models for the rare reversions within 10Kb of HAQERs, HARs, or RAND (random matched) sequences, while the ranges represent the 95% confidence interval. Solid points indicate p-value < 0.05. **D-F** Distributions of rare reversions counts from the SPARK whole genome sequencing data within 10Kb of the following regions: HAQERs (D), HARs (E), and random sequence (RAND, F).

We validated HAQER effects on language across multiple independent cohorts using both common and rare genetic variants. In the SPARK autism dataset (N > 30,000) (36), HAQER CP-PGS predicted verbal language capability (”Able to talk using short phrases or sentences”, ES-PGS *β* = 0.05, p-value = 0.008, N = 29,266) and language disorder diagnoses in parents without autism (model improvement p-value = 7.9 × 10^−5^, N = 713) but not psychiatric conditions (model improvement p-value = 0.58, N = 713), confirming language specific effects (Figure 4A–B, Supplementary Table 5). Clinical records showed HAQER CP-PGS is associated with verbal IQ (ES-PGS *β* = 2.08, p-value = 0.022, N = 620) but not nonverbal IQ (ES-PGS *β* = 0.59, p-value = 0.49, N = 620, Figure S3), further supporting specificity.

To test HAQER effects through an orthogonal approach independent of our ES-PGS method, we analyzed rare genetic variation in SPARK whole genome sequencing data (N > 2,000). We examined rare ”reversions”, variants that revert from the human-specific version to their human-chimp ancestral state, reasoning that if HAQERs evolved to support human language, reversions should impair language function, which would support the observed polygenic score associations. Individuals carrying more reversions in HAQERs showed increased likelihood of developmental language disorder (*β* = 0.16, p-value = 6.5 × 10^−4^) and delayed language developmental milestones, but no association with age started walking or intellectual disability (Figure 4C, Supplementary Table 6). Notably, HAQERs showed higher rates of reversions compared to HARs and matched random sequences (Figure 4D–F).

Further independent validation in the ABCD developmental cohort (37) showed the HAQER CP-PGS is associated with the Rey Auditory Verbal Learning Test performance, a measure of spoken word recall (ES-PGS *β* = 0.24, p-value = 0.048, N = 5,625), but not reading or vocabulary tasks from the NIH Toolbox (Supplementary Table 7). This suggests HAQERs have preferential effects on vocal communication over written language. These converging results across cohorts and variant types strongly support that genetic variation within HAQERs have a significant and specific effect on spoken language abilities in contemporary humans.

### HAQERs evolved stronger binding affinity for language-relevant transcription factors

To investigate the molecular mechanisms underlying HAQERs’ association with language development, we analyzed how rare genetic variants affect transcription factor binding sites in these regions. We compared two classes of rare variants in the EpiSLI cohort: hominin-chimpanzee ancestral allele reversions versus other rare variants, using position weight matrices to quantify how these variants alter predicted transcription factor binding affinity. By comparing the effects of reversions with other rare variants, we could detect systematic evolutionary changes in hominin-specific transcription factor binding associated with language phenotypes.

**Figure 5:**
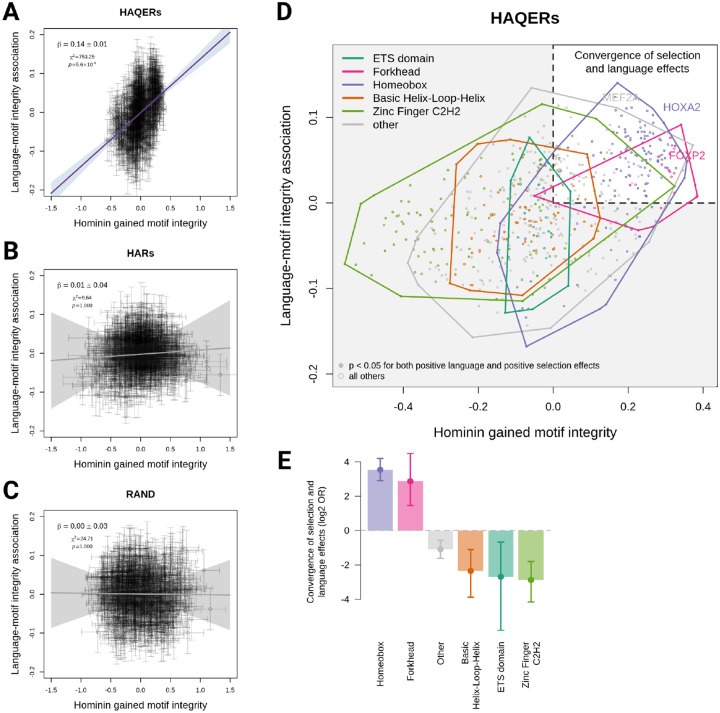
Hominin gained transcription factor binding in HAQERs influences language **A-C** Relationship between selection for transcription factor motif integrity (x-axis) and motif association with language ability (y-axis) in (A) HAQERs, (B) HARs, and (C) random genomic regions. Each point represents one transcription factor motif. Error bars indicate ±1 standard error. Purple line (or gray for non-significant fits) shows York regression fit with 95% confidence interval (shaded); regression coefficient (*β*), chi-squared statistic (*χ*^2^), and p-values are shown. **D** Detailed view of motif effects in HAQERs colored by transcription factor family. Solid points indicate motifs with p < 0.05 for both positive selection and positive language association. Colored polygons show convex hulls for each transcription factor family. **E** Enrichment analysis of transcription factor families for concordant positive selection and language effects, shown as log2 odds ratios. Error bars indicate 95% confidence intervals. Solid points indicate p < 0.05.

In HAQERs, hominin-gained transcription factor motif binding showed significant correlation with individual core language ability (N = 350 individuals, *β* = 0.14, p-value = 5.6×10^−4^, Figure 5A), indicating that hominins evolved increased transcription factor binding in these regions and this binding enhances language performance. In contrast, regions under sequence conservation with human-specific changes (HARs, *β* = 0.01, p-value = 1, Figure 5B) or neutral evolution (RAND sequences, *β* = 0, p-value = 1, Figure 5C) showed no relationship between motif integrity and language ability, highlighting HAQERs’ unique and systematic selection for regulatory function during hominin evolution.

Analysis of specific transcription factor families revealed striking enrichment of Homeobox and Forkhead box transcription factors associated with both enhanced binding affinity in HAQERs and improved language performance (Figure 5D). The Homeobox family displayed the strongest enrichment among all transcription factor families (odds ratio = 11.58, p-value = 2.6 × 10^−34^), followed by the Forkhead box family (which includes *FOXP2*, odds ratio = 7.28, p-value = 5.3 × 10^−6^, Figure 5E, Supplementary Table 16). These results suggest that hominin-gained binding of Homeobox and Forkhead box families within HAQERs may have played a crucial role in the evolution of human language capability.

### HAQERs regulate language-relevant brain circuits through human-specific chromatin accessibility

To determine which brain cell types are regulated by HAQERs, we analyzed their overlap with candidate cis-regulatory elements (cCREs) identified through single-nucleus chromatin accessibility profiling (snATAC-seq) of human and mouse brain cell types (38). We tested whether HAQERs preferentially associate with human-specific versus evolutionarily conserved chromatin accessible regions, reasoning that HAQERs should show increased overlap with human-specific regulatory elements if they provide novel functions in the human lineage.

HAQERs demonstrated significant enrichment for human-specific cCREs across brain cell types (Figure S5A), with the strongest enrichment in medium spiny neurons (MSNs, p-value = 9.8 × 10^−8^). MSNs comprise over 90% of neurons in the human striatum, a circuit that plays essential roles in vocal learning across species (17, 39) and was the only part of the brain robustly associated with developmental language disorders in a recent meta-analysis (40). HAQERs also showed enrichment around human-specific cCREs in *FOXP2*-expressing neurons (p-value = 5.3 × 10^−4^), providing independent evidence linking HAQERs to *FOXP2* regulatory networks beyond our transcription factor binding findings (Figure 5E). In contrast, HARs showed minimal enrichment for human-specific chromatin accessibility but strong enrichment for evolutionarily conserved regions (strongest in VIP neurons, p-value = 2.7 × 10^−10^, Figure S5B). These results are consistent with HAQERs providing novel regulatory functions specific to human brain development that may support language capabilities.

### Selective pressures acting on language and general cognition

**Figure 6:**
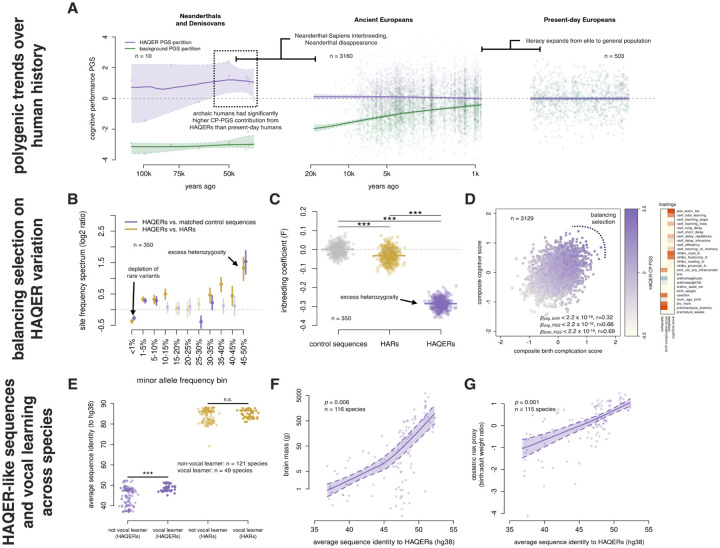
Selective pressures on human cognition and convergent evolution of vocal learning **A** CP ES-PGS across hominin evolution. HAQER PGS (purple) and background PGS (green) plotted against sample age for Neanderthals/Denisovans, Ancient Europeans, and Present-day Europeans. LOESS fits with 95% CIs shown. **B** Site frequency spectrum comparing HAQERs to matched controls and HARs. *Log*_2_ ratio of proportion of variants across allele frequency bins; positive values indicate HAQER enrichment. Error bars: 95% bootstrap CIs. **C** Inbreeding coefficient (F-statistic) across sequence types. Lower F-statistics indicate excess heterozygosity. HAQERs show significantly lower F-statistics than HARs and control regions (*** indicates p-value < 0.001). **D** Composite phenotype analysis linking HAQER CP-PGS to birth complications and cognitive scores. Scatterplot: canonical correlation components colored by HAQER CP-PGS. Heatmap: variable loadings showing birth complications on component 1, cognitive variables on component 2. **E** HAQER-like and HAR-like sequence similarity scores in non-vocal learning (N = 121 species) and vocal learning (N = 49 species) mammals. Phylogenetic logistic regression statistics are shown. **F-G** HAQER-like sequence similarity correlates with brain mass (N = 116 species, **F**) and birth:adult weight ratio (N = 115 species, **G**) across mammals. LOESS fits with 95% CIs.

Having multiple lines of evidence associating HAQERs with human language evolution, we next examined how selective pressures may have influenced language-related genetic variation over the past 20,000 years of human history using the Allen Ancient DNA Resource (AADR) (41). The AADR is the largest genotyped collection of ancient humans, providing harmonized genotype and metadata for each sample (like radiocarbon dating based sample ages). We identified ancient west Eurasians, then correlated their HAQER CP-PGS and the background CP-PGS with sample age (N = 3,244 individuals with remains dated between 18,775 to 150 years ago passing quality control). We see that the polygenic score for general cognition (background CP-PGS) has been subject to positive selection and has increased substantially over time (selection coefficient = 0.088, p-value = 2.1 × 10^−12^), Figure 6A. Unexpectedly, we found that HAQER CP-PGS has been stable throughout human history, indicating that ancient and modern humans carry similar numbers of language-related alleles in HAQERs (selection coefficient = −0.004, p-value = 0.71, Figure 6A).

The presence of HAQERs in archaic humans provided a unique opportunity to describe genetically predicted cognitive traits across human species. To do this, we computed HAQER CP-PGS and background CP-PGS in archaic humans (N = 10) and compared them to ancient (N = 3,244) and modern humans (N = 503). Remarkably, the ten archaic human genomes (eight Neanderthals and two Denisovans) showed elevated HAQER CP-PGS (mean z-score = 0.91, median z-score = 1.23), while having reduced background CP-PGS (mean z-score = −3.02, median z-score = −3.01, Figure 6A). In contrast, a set of random matched control regions showed no differences in CP-PGS across groups (Supplementary Figure S4A–C, Supplementary Table 9). While this data should be interpreted with caution due to challenges applying polygenic scores across populations (42), the elevated HAQER CP-PGS we observed aligns with arguments that archaic humans were capable of complex language (43–45).

### Evidence of balancing selection from modern genomes

The stability of HAQER CP-PGS throughout human evolutionary history led us to hypothesize that HAQERs have been maintained through balancing selection. Multiple population genetic analyses in the EpiSLI sample provided support for this hypothesis. HAQERs exhibited significantly more heterozygosity compared to both HARs (t-statistic = 110, p-value = 5.3×10^−273^) and matched control sequences (t-statistic = 147, p-value = 1.6 × 10^−315^), suggesting that heterozygosity at HAQER loci provided a selective advantage (Figure 6C). Additionally, we observed an enrichment of intermediate frequency variants (MAF 30–50%) in HAQERs (Figure 6B). Both excess heterozygosity and enrichment of intermediate allele frequencies are characteristic signatures of balancing selection. Together with our ancient DNA results, these findings support a model where selective pressures prevented fixation of language-enhancing alleles in HAQERs, maintaining genetic variation at intermediate frequencies throughout human history.

### HAQERs influence prenatal brain development

The evolutionary analysis revealed a puzzling pattern: while other cognitive variants show recent positive selection, language-promoting HAQER variants have remained stable for at least the past 20,000 years of human history despite their clear cognitive benefits. This stability suggests ongoing fitness costs that counterbalance the advantages of enhanced language ability. Given HAQERs’ established role in neurodevelopment (34), we investigated whether these variants create pleiotropic effects on prenatal brain development that could explain their evolutionary constraints. First, we investigated temporal and cell-type enrichment for these regions to identify if they were likely to influence birth related neurodevelopmental traits. HAQERs showed broad enrichment for variants affecting prenatal brain gene expression when intersected with single-cell quantitative trait loci (scQTLs) from developing midbrain neurons (46). The strongest enrichment observed was at the late prenatal time point, which corresponds to when the human brain most rapidly expands (47) (Figure S5C). Critically, HAQERs showed no enrichment when we examined adult brain regulatory elements (48), confirming prenatal neurodevelopmental effects (Figure S5D). HAQERs were also significantly enriched around genomic loci associated with head circumference at birth, a proxy for brain size (49) (p-value = 4.4 × 10^−4^, Figure S5E).

### HAQERs link language evolution to the obstetric dilemma

The evidence for HAQER effects on prenatal brain development and head circumference at birth suggests a potential mechanism for their evolutionary stability: the obstetric dilemma. Enhanced fetal brain development may create reproductive costs through the obstetric dilemma, where increased brain size complicates birth in bipedal humans with narrow pelvises (50, 51). To test whether HAQERs contribute to this trade-off, we analyzed brain imaging, cognitive, and birth outcome data in the ABCD cohort (37).

A canonical correlation analysis revealed two distinct composite phenotypic axes associated with HAQER CP-PGS, providing evidence for the obstetric dilemma that could plausibly drive the observed balancing selection (Figure 6D). The first canonical component captured variance primarily from birth complication-related variables, while the second component loaded predominantly on cognitive performance measures (including a measure of verbal language learning). Critically, both composite scores showed positive correlations with HAQER CP-PGS (*r* = 0.69 and *r* = 0.66 respectively; p-value < 2.2 × 10^−16^ for both), and the two composite scores were themselves positively correlated (*r*=0.32, p-value < 2.2 × 10^−16^). This pattern indicates that genetic variants contributing to higher HAQER CP-PGS simultaneously increase both cognition (a trait under positive selection) and birth complication risk (a trait under negative selection). The consistent positive relationship between HAQER CP-PGS and both phenotypic domains provides a plausible mechanistic explanation for the balancing selection signatures specific to HAQERs. While the correlation magnitudes should be interpreted cautiously given our optimization procedure (see Materials and Methods), the qualitative finding of antagonistic pleiotropy is robust and aligns with the evolutionary hypothesis that the cognitive benefits conferred by HAQER variants are counterbalanced by obstetric costs, maintaining genetic diversity at these loci through balancing selection. This trade-off between cognitive ability and increased birth complications is a fundamental constraint that may have shaped the evolution of human language.

### HAQER-like sequences show convergent evolution in vocal learning mammals

To test whether HAQER functions extend beyond humans, we analyzed homologous sequences across 170 non-primate mammalian species, including 49 vocal learners and 121 non-vocal learner species (15). Vocal learner species can acquire and modify vocalizations through experience, contrasting with species restricted to innate vocalizations. We computed genome-wide ”HAQER-like” and ”HAR-like” sequence similarity scores using multiple sequence alignments (52) and tested for associations with vocal learning ability while controlling for phylogenetic relatedness (53, 54).

Vocal learner species showed significantly higher HAQER-like sequence similarity compared to non-vocal learners (phylogenetic regression *β* = 1.41, p-value = 1 × 10^−4^, Figure 6E). HAQERs were also enriched around previously identified mammalian vocal learner enhancer regions (p-value = 0.028, Figure S5F) (15). While HARs showed a similar enrichment around vocal learner enhancer regions (p-value = 0.04), HAR sequence similarity was not associated with vocal learner classification (phylogenetic regression *β* = −0.15, p-value = 0.67, Figure 6E). Given the independent evolution of vocal learning across mammalian lineages, these results suggest HAQER-like sequences may be a fundamental genetic mechanism for complex vocal communication that has been repeatedly utilized across evolutionary history.

Remarkably, HAQER-like sequences also associated with brain size across species (phylogenetic regression *β* = 0.42, p-value = 0.006, N = 116 species) and larger relative birth weights (phylogenetic regression *β* = 0.44, p-value = 0.001, N = 115 species), mirroring the human obstetric dilemma pattern (Figure 6F–G). This convergent evidence across independent evolutionary lineages supports a link between the genetic architecture of vocal learning, brain development, and reproductive constraints. Together, these results suggests the trade-offs we observe in human language evolution may represent a broader biological phenomenon that support complex vocal communication.

## Discussion

Our evolutionary stratified polygenic score analysis identifies genomic regions that disproportionately contributed to human language evolution and continue to influence individual differences in language abilities observed in present-day humans. While previous research demonstrated that rare mutations in *FOXP2* can cause language disorders (5, 55), common variants in *FOXP2* show minimal association with typical language variation (7, 8), prompting GWAS studies and polygenic models of language-related traits (9, 11–14, 56). However, these models left critical questions unanswered: when did language-associated variation evolve, and how do these molecular changes influence language development? Our analysis reveals that HAQERs (31, 34), mostly non-coding regions that rapidly evolved before the human-Neanderthal split and represent less than 0.1% of the human genome, harbor variants with disproportionate effects on language. Individual SNPs in HAQERs carry 188 times more impact on language than variants elsewhere in the genome, while showing no association with nonverbal cognition. These potent regulatory effects distributed across thousands of small genomic elements support the polygenic architecture of human language while pointing to its specific evolutionary origins.

We find that HAQERs evolved across hominins to increase binding for Forkhead (including *FOXP2*) and Homeobox transcription factors (TFs), with motif integrity correlating with individual language scores. Supporting this regulatory mechanism, HAQERs show significant enrichment for human-specific chromatin accessible regions across brain cell types, with strong enrichment in medium spiny neurons and *FOXP2*-expressing neurons, while HARs primarily overlap with evolutionarily conserved regulatory elements. This suggests *FOXP2* influences language primarily through its regulatory networks (57) rather than protein-coding changes, explaining how rare mutations in a single TF can produce profound effects on language development while common variants in the same TF shows minimal associations (7, 8). The observed 11.58-fold and 7.28-fold enrichment of hominin-gained Homeobox and Forkhead binding sites that positively correlate with language scores in HAQERs indicates that these developmentally essential TF families (58–60) likely became central to human language evolution.

Cross-species triangulation provides independent support that HAQERs are functional regulatory elements for language-related traits. Consistent with our human evidence, vocal learner mammals show significantly higher HAQER-like sequence similarity than non-vocal learners after controlling for phylogenetic relationships, with parallel associations for brain size and birth weight. Importantly, HAQERs show enrichment around established mammalian vocal learning enhancer regions (15), consistent with previous reports of convergent evolution for complex vocal communication (15–20). This convergent evolution of HAQER-like sequences across vocal learning lineages provides strong independent support that these regulatory elements are fundamental genetic mechanisms for complex vocal communication.

Ancient DNA analysis reveals that while general cognitive variants show positive selection over 20,000 years, language-related HAQER variants have remained stable, suggesting balancing selection maintains language-related genetic variation. Analysis of birth outcomes, brain imaging, and cognitive data provided a potential mechanism for this unexpected evolutionary constraint: individuals with more language-related variants in HAQERs were more likely to have larger heads and birth complications, indicating trade-offs between language capability and reproductive costs. This pattern connects HAQERs to the obstetric dilemma, the evolutionary trade-off between narrower pelvises supporting upright walking and larger fetal brains enabling complex cognition (50, 51), potentially explaining why language-promoting variants persist at intermediate frequencies rather than reaching fixation. Further supporting this neurodevelopmental mechanism, we find that HAQERs are enriched for genetic variants associated with prenatal gene expression and head circumference at birth (46, 49). The prenatal brain regulatory activity of HAQERs aligns with established evidence that early developmental processes critically influence later language outcomes (61, 62). Intriguingly, despite substantial methodological limitations in cross-population polygenic score applications (42), available Neanderthal and Denisovan genomes suggest higher frequencies of language-promoting HAQER variants than modern humans, though this requires cautious interpretation. This finding needs further validation, and future research should also investigate morphological and obstetric differences between archaic and modern humans that may have enabled archaic populations to carry higher polygenic scores of language-associated alleles in HAQERs. These results point to the obstetric dilemma as an ongoing evolutionary constraint that specifically limits language-related genetic variants from reaching fixation, creating a fundamentally different selection landscape for vocal communication compared to general cognitive abilities.

These findings demonstrate how ancient regulatory innovations continue to shape human language variation through evolutionary constraints that balance cognitive benefits against reproductive costs. The independent evolution of HAQER-like sequences in vocal learning mammals reveals fundamental genetic mechanisms for complex communication that have emerged repeatedly across lineages. While general cognitive variants show positive selection over time, language variants remain stable at intermediate frequencies, suggesting that individual differences in spoken language abilities reflect ongoing evolutionary trade-offs. These evolutionary constraints reveal why individual differences in language abilities persist despite the importance of communication skills. Further investigation of HAQER regulatory networks will be essential to translate these evolutionary insights into approaches for supporting those with language disorders.

## Materials and methods summary

We developed an evolutionary stratified polygenic score (ES-PGS) approach to trace the origins of language-relevant genetic variation across 65 million years of primate evolutionary events. Language abilities were assessed through factor analysis of 17 longitudinal cognitive and language assessments from kindergarten through 4th grade in 350 children from a community-based cohort (EpiSLI) (21). We applied ES-PGS using cognitive performance (28) polygenic scores partitioned across five evolutionary annotations (29–33), testing whether genomic regions from specific evolutionary periods contribute disproportionately to language versus general cognitive functions.

Validation was performed across multiple independent cohorts using both common and rare genetic variants. In the SPARK autism dataset (N > 30,000) (36), we tested HAQER effects on verbal language ability and language disorder diagnoses. We analyzed rare ”reversions” in SPARK whole genome sequencing data (N > 2,000 individuals), variants reverting from human-specific to human-chimpanzee ancestral states (34), reasoning that if HAQERs evolved to enhance language, reversions should impair language function. Additional validation used unrelated individuals from the ABCD cohort (N = 5,625) for spoken word recall performance, cognitive test scores, brain size, and birth traits (37).

To investigate molecular mechanisms supporting language evolution, we analyzed how rare genetic variants in evolutionarily significant regions affect transcription factor binding sites using position weight matrices for 633 human transcription factors from JASPAR2020 (63). We compared hominin-chimpanzee ancestral allele reversions versus other rare variants to detect systematic evolutionary changes in transcription factor binding associated with language phenotypes. Additionally, we looked for enrichment of evolutionarily significant regions across human-specific or conserved chromatin accessible regions (38), birth head circumference GWAS loci (49), neurodevelopmental related scQTLs datasets (46, 48), and mammalian vocal learning genomic regions (15).

Ancient DNA analysis examined selective pressures using the Allen Ancient DNA Resource (41), correlating HAQER polygenic scores with sample age in 3,244 ancient west Eurasians (18,775–150 years ago). We computed polygenic scores in archaic humans (8 Neanderthals, 2 Denisovans) and compared them to ancient and modern humans. Cross-species validation analyzed HAQER-like sequence similarity across 170 non-primate mammalian species (49 vocal learning, 121 non-vocal learning) (15) using phylogenetic regression to control for evolutionary relatedness (53, 54) in analyses of vocal learning, brain size, and a birth:adult weight ratio (used as a rough proxy for the obstetric dilemma) (64).

## Supplementary Material

Supplement 1

1

Materials and Methods

Figs. S1 to S5

References (65–141)

## Figures and Tables

**Figure 1:**
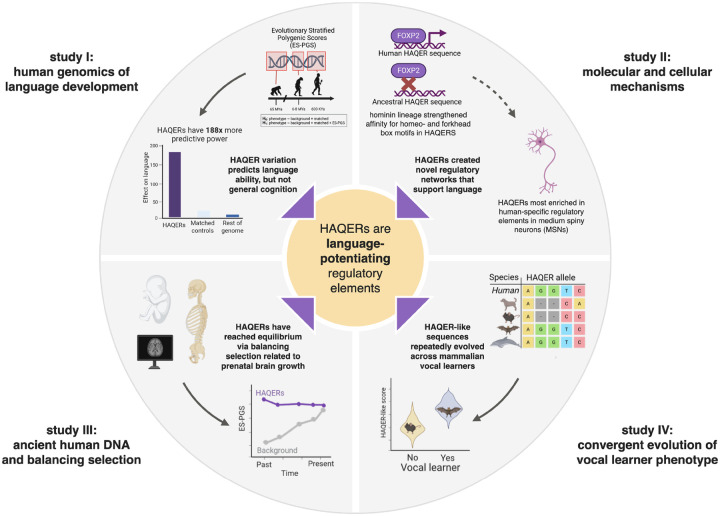
Overview of this study and key findings

## Data Availability

Custom code for ES-PGS and all analyses in this paper is available here, including example code for ES-PGS that can be applied to other research problems: https://github.com/lucasgcasten/language_evolution The EpiSLI whole genome sequencing data described here is available to qualified researches via dbGaP (study accession = phs002255.v1.p1): https://www.ncbi.nlm.nih.gov/projects/gap/cgi-bin/study.cgi?study_id=phs002255.v1.p1 SPARK genetic and phenotype data is available to qualified researchers at SFARI base: https://base.sfari.org/ ABCD is available to qualified researchers at: https://nda.nih.gov/abcd/request-access 1000 Genomes Phase 3 data is available at: https://www.internationalgenome.org/data/ Allen Ancient DNA Resource: https://reich.hms.harvard.edu/allen-ancient-dna-resource-aadr-downloadable-genotypes-present-Neanderthal Neanderthal and Denisovan genomes: https://www.eva.mpg.de/genetics/genome-projects/ Cross-species sequence alignment data: https://hgdownload.soe.ucsc.edu/goldenPath/hg38/cactus447way/ PanTHERIA: https://figshare.com/articles/dataset/Full_Archive/3531875 We used publicly available tools for data processing and analysis: bcftools: https://samtools.github.io/bcftools/bcftools.html PLINK: https://www.cog-genomics.org/plink GCTA: https://yanglab.westlake.edu.cn/software/gcta LDpred2: https://privefl.github.io/bigsnpr/articles/LDpred2.html PRSet: https://choishingwan.github.io/PRSice/quick_start_prset/ bedtools: https://bedtools.readthedocs.io Biopython: https://biopython.org/ gonomics: https://github.com/vertgenlab/gonomics R: https://www.r-project.org/
