# Supplementary material for: Ancient regulatory evolution shapes individual language abilities in present-day humans
Source: bioRxiv. 2026 Feb 13:2025.03.07.641231. Originally published 2025 Mar 10. Preprint. [Version 4] doi: 10.1101/2025.03.07.641231 (PMC11952349; doi:10.1101/2025.03.07.641231)
Supplement: 1 [file NIHPP2025.03.07.641231V4-supplement-1.pdf]

bioRxiv preprint doi: https://doi.org/10.1101/2025.03.07.641231; this version posted February 14, 2026. The copyright holder for this preprint (which was not certified by peer review) is the author/funder, who has granted bioRxiv a license to display the preprint in perpetuity. It is made available under aCC-BY 4.0 International license.

Supplementary Materials for

Ancient regulatory evolution shapes individual language abilities in present-day humans

Lucas G. Casten†,Tanner Koomar†,Taylor R. Thomas,Jin-Young Koh,Dabney Hofammann,Savantha Thenuwara,Allison Momany,Marlea O'Brien,Jeff C. Murray,J. Bruce Tomblin,Jacob J. Michaelson*

*Corresponding author. Email: jacob-michaelson@uiowa.edu

†These authors contributed equally to this work.

This PDF file includes:

Materials and Methods

Figures S1 to S5

bioRxiv preprint doi: https://doi.org/10.1101/2025.03.07.641231; this version posted February 14, 2026. The copyright holder for this preprint (which was not certified by peer review) is the author/funder, who has granted bioRxiv a license to display the preprint in perpetuity. It is made available under aCC-BY 4.0 International license.

Materials and Methods

Human Subjects and IRB

All EpiSLI subjects in this study were minors who assented to participation under the University of Iowa IRB# 200511767. Secondary genetic analyses for EpiSLI subjects were approved and carried out under the University of Iowa IRB #201406727. SPARK is approved under WIRB #201703201. Approval for data collection and analysis of the SPARK Research Match study described here was conducted under the the University of Iowa IRB #201705739.

EpiSLI Cohort

The discovery cohort of this study, referred to as EpiSLI, was originally recruited as an epidemiological study of language impairments in Kindergarteners in the state of Iowa. A more detailed description of the recruitment scheme can be found in the initial (*65*), and subsequent (*21, 66*) publications. In brief, 7,218 kindergarteners, sampled to be representative of Iowa's population were screened for language impairment with a rapid, 40-item subset (*65*) of the TOLD-2P (*67*). Children who failed the TOLD-2P screener (i.e., have poor language ability) were over-sampled to capture a broad range of language ability in the primary cohort (n = 1,929 children). Children with autism spectrum disorder and/or intellectual disability were excluded. The entirety of the primary cohort received a more complete battery of language and cognitive assessments, while teachers and parents completed scholastic and behavioral questionnaires about the children 2nd, 4th, 8th, and 10th grades. Language and cognitive assessments included: TOLD-2P (*67*), WPPSI (*68*), a narrative story task (*69*), Woodcock Reading Mastery Tests-Revised (*70*), Word-Sound Deletion task (*71*), Random Animals-Colors task (*71*). We selected a total of 390 children from the primary cohort for whole genome sequencing. This sample represents a broad spectrum of language abilities, these 390 children were chosen by sampling from the tails of the distribution of a composite language score inward until we reached our final sample size (*8, 65*).

Behavioral assessment scores came from normed T-scores for the summary scales of the Child Behavior Checklist (CBCL) developed by ASEBA (*72*). CBCLs were completed at the 2nd grade timepoint of the study (241 of the 350 children used in this study had complete CBCL data for the analysis in Supplementary Table 2).

bioRxiv preprint doi: https://doi.org/10.1101/2025.03.07.641231; this version posted February 14, 2026. The copyright holder for this preprint (which was not certified by peer review) is the author/funder, who has granted bioRxiv a license to display the preprint in perpetuity. It is made available under aCC-BY 4.0 International license.

Factor Analysis

An exploratory maximum-likelihood factor analysis was carried out on the language and cognitive assessment scores using the `factanal` function from the `stats` package in R (*73*), with the default "varimax" rotation. A number of factors from 1-10 were evaluated, with 7 - the largest number of factors with a nominal χ^2 p-value less than 0.05 - chosen for subsequent analyses (Figure **??**A). These seven factors accounted for an estimated 62.3% of the total variance in the original data.

EpiSLI Cognitive measures

Core language assessment scores and IQ were normalized via the general scheme described in (*65*) to account for the over-sampling of individuals with low language ability. Core language scores were corrected for age as the residual of a linear regression model using `lm()` in R (*73*). The age-corrected core language scores were then normalized with respect to the population using the `wtd.mean()` and `wtd.var()` functions from the `Hmisc` package (*74*) in R.

Cognitive data Imputation

Most individuals for whom whole genome sequencing was generated had scores from assessments given in 2nd grade, while a minority had only 4th grade (41/390) or kindergarten (39/390) scores. Missing scores were imputed using chained random forests and predictive mean matching as implemented the `missRanger` R package (*75*) with 5,000 trees and `pmm.k = 20`.

EpiSLI Whole Genome Sequencing

Sample Collection and whole genome sequencing

DNA was collected and extracted from either blood or saliva samples for 390 individuals (350 of which were used in the final analysis after quality control). DNA concentration of all sequenced samples was quantified with with Qubit 2.0 Fluorometer (Life Technologies Corporation). 390 DNA samples were sheared on a E220 Focused-ultrasonicator (Covaris) to an average size of 400 bp. Sequencing libraries were generated with a Kapa Hyper Prep kit (Kapa Biosystems) according to the manufacturer protocol. All samples had an average genome-wide coverage of at least 20X and were sequenced on a HiSeq4000 (Illumina) with 150-bp Paired End chemistry.

bioRxiv preprint doi: https://doi.org/10.1101/2025.03.07.641231; this version posted February 14, 2026. The copyright holder for this preprint (which was not certified by peer review) is the author/funder, who has granted bioRxiv a license to display the preprint in perpetuity. It is made available under aCC-BY 4.0 International license.

Post-sequencing QC

All sequencing data was analysed with Fastqc (v0.11.8) (*76*), where no samples failed the run modules. After genome alignment (see below), some samples were found to have significantly better coverage than others, in excess of 40x. To help control for ascertainment bias in these samples, all samples with an average coverage > 35x (based on initially mapped BAM) were randomly down-sampled, leading to the distribution of genome-wide coverage and average insert-size outlined in Supplementary Table 17.

Genome Alignment and Variant Calling

Reads were processed with bcbio (v1.1.6) (*77*) and mapped to hg19 via BWA-mem (v0.7.17) (*78*). SNVs and InDels were then called with all samples in a pool with three variant callers: GATK (v4.1.2.0) (*79*), FreeBayes (v1.1.0.46) (*80*), and Platypus (v0.8.1.2) (*81*).

Variants from each caller were filtered according to caller-specific quality metrics. For GATK, variants needed to pass all VQSR tranche thresholds. For Platypus, variants were filtered based on the goodness of fit of genotype calls, excessive region-based haplotype scores, root-mean-square mapping quality, variant quality and its ratio with read depth, low complexity sequence context, allele bias, region-based read quality, neighboring homopolymers, and strand bias. For FreeBayes, variants were filtered based on a combination of allele frequency, read depth, and overall quality. The thresholds used for filtering Platypus and FreeBayes calls were the default set by bcbio. An ensemble of all three callers was generated and used in all subsequent analyses in order to achieve improved specificity in the detection of rare variants. All variants in the ensemble callset were called by GATK as well as either Platypus OR FreeBayes. The majority of variants (87%; 25,775,508) were called by all three callers, while a minority were called by GATK and only one other caller (13%; 3,937,146). All variants were filtered to have a QUAL and individual quality score ≥ 20. Qualified researchers can access the individual level genotypes from dbGaP at (study accession = phs002255.v1.p1): `https://www.ncbi.nlm.nih.gov/projects/gap/cgi-bin/study.cgi?study_id=phs002255.v1.p1`

bioRxiv preprint doi: https://doi.org/10.1101/2025.03.07.641231; this version posted February 14, 2026. The copyright holder for this preprint (which was not certified by peer review) is the author/funder, who has granted bioRxiv a license to display the preprint in perpetuity. It is made available under aCC-BY 4.0 International license.

Final sample QC

Starting from the initial sample size of 390, a total of 14 samples were flagged for removal from all association analyses because of population stratification. Specifically, these samples did not cluster with 1,000 Genomes Europeans (*82*), or were more than 3 standard deviations away from the rest of the EpiSLI cohort based on the top 10 multidimensional scaling components calculated from SNPs found at or above a 0.05 minor allele frequency. An additional 26 samples were dropped due to relatedness or limited phenotypic data, leaving a final sample size of 350 unrelated European individuals with complete data for all genomic analyses.

Variant Annotations

Variants were annotated with the Ensembl Variant Effect Predictor tool (VEP v109) (*83*). Reference population allele frequencies came from 1000 Genomes Phase 3 samples (*82*) and GnomAD (*84*), rsIDs of variants were dbSNP (*85*) (v151), these additional annotations were added using VCFanno (*86*) (v20190119).

Polygenic Scores (PGS)

Genotypes for PGS

To derive a set of SNPs suitable for PGS analysis, we merged our dataset with 1000 Genomes Europeans to use as a reference sample. Based on widely used recommendations (*87*), we extracted SNPs with a minor allele frequency $\geq 1\%$, Hardy-Weinberg equilibrium p-value $> 1 \times 10^{-6}$, and a missingness rate $< 2\%$ in both samples (leaving 7,719,665 SNPs total for PGS calculation).

PGS Calculation

LDpred2 was used to calculate a genome-wide PGS for all traits with the infinitesimal model using the provided UK Biobank LD reference panel and HapMap3+ variant set (*88*). PGS were calculated using GWAS summary statistics for neurodevelopmental and psychiatric traits: ADHD (*89*), addiction (*90*), alcohol dependency (*91*), Alzheimer's (*92*), Autism (*93*), Anorexia (*94*), bipolar disorder (*95*), cannabis use disorder (*96*), depression (*97*), epilepsy (*98*), insomnia (*99*), neurodevelopmental conditions (*100*), PTSD (*101*), schizophrenia (*102*), and Tourette's (*103*).

bioRxiv preprint doi: https://doi.org/10.1101/2025.03.07.641231; this version posted February 14, 2026. The copyright holder for this preprint (which was not certified by peer review) is the author/funder, who has granted bioRxiv a license to display the preprint in perpetuity. It is made available under aCC-BY 4.0 International license.

GWAS summary statistics for cognitive traits included: cognitive performance (*104*), educational attainment (*105*), executive functioning (*106*), and the "g Factor" (*107*). Additional PGS were calculated for the following behavioral and socioeconomic status related traits: childhood aggression (*108*), antisocial behavior (*109*), empathy (*110*), the BIG5 personality traits (*111*), income (*112*), and the Townsend Deprivation Index (a measure of material deprivation) (*113*). PGS were also calculated for brain structural (*114, 115*) and functional connectivity phenotypes (*116*). Finally, we computed PGS for miscellaneous traits: left-handedness (*113*), height (*117*), childhood trauma (*118*), and vocal pitch (*119*). It is important to note we did not compute PGS in EpiSLI for reading based traits, because our sample was part of the discovery cohort of the largest reading related GWAS to date (*9*). Associations for all of these PGS with our language factors can be found in Supplementary Table 3.

To account for population stratification, we corrected PGS for the first 5 genetic principal components. PGS were normed to the 1000 Genomes Europeans reference sample.

ES-PGS Calculation

We developed evolutionary stratified polygenic scores (ES-PGS) to identify genomic regions from specific evolutionary periods that disproportionately contribute to modern phenotypic variation. ES-PGS partitions polygenic score effects based on evolutionary annotations, testing whether variants from particular evolutionary epochs show disproportionately strong associations with traits compared to the broader genome.

The method uses three components: (1) annotation-specific PGS calculated from variants within evolutionary regions of interest, (2) background PGS calculated from all remaining genome-wide variants, and (3) matched control PGS from biologically similar regions to test specificity. Matched control regions were generated by sampling 1,000 random genomic segments for each annotation region (e.g., 10 annotation region would produce 10,000 random regions for matching), these regions are then matched for chromosome, size, GC content, repeat content, distance to nearest gene, and overlap with promoter or coding sequences. Control regions were required to be *geq* 100kb from any annotation region to ensure independence.

We implemented ES-PGS using nested model comparisons. The reduced model tests background genomic effects and matched control effects:

bioRxiv preprint doi: https://doi.org/10.1101/2025.03.07.641231; this version posted February 14, 2026. The copyright holder for this preprint (which was not certified by peer review) is the author/funder, who has granted bioRxiv a license to display the preprint in perpetuity. It is made available under aCC-BY 4.0 International license.

$$y = \beta_0 + \beta_1 \text{PGS}_{\text{background}} + \beta_2 \text{PGS}_{\text{matched}} + \epsilon \tag{S1}$$

The full model adds the annotation-specific term:

$$y = \beta_0 + \beta_1 \text{PGS}_{\text{background}} + \beta_2 \text{PGS}_{\text{matched}} + \beta_3 \text{PGS}_{\text{annotation}} + \epsilon \tag{S2}$$

Where y represents the phenotype (e.g., language factor scores), $\text{PGS}_{\text{background}}$ includes all variants except those in the focal annotation, $\text{PGS}_{\text{matched}}$ represents the matched control regions, and $\text{PGS}_{\text{annotation}}$ contains only variants within the evolutionary annotation. An ANOVA comparing these models tests whether the annotation contributes significantly beyond background genetic effects and matched controls. Additional covariates (genetic principal components, demographics) can be incorporated as needed.

PRSet (*27*) was used to calculate stratified PGS using human genome annotations in BED formatted files, computing a clumping and thresholding based ES-PGS for each annotation and a background PGS for the rest of the genome. Background regions for each annotation were identified using the ”complement” function from bedtools (v2.26.0) (*120*). Matched control regions were matched using the `nullranges` R package (*121*). As recommended by the authors of PRSet, we used a p-value threshold of 1 and the 1000 Genomes Europeans as the LD reference (*82*). We corrected for population stratification using the same steps as described in the genome-wide PGS analysis.

We focused our analysis on genome annotations related to primate and human evolution, annotations included: primate ultra conserved regions (primate UCEs, Supplementary Data 5 from the reference) (*29*), great ape lineage accelerated regions (*30*)(Supplementary Data S1 filtered to ”Hominidae” from the reference), Human Ancestor Quickly Evolved Regions (HAQERs, Supplementary Table 68 from the reference) (*31*), Human Accelerated Regions (HARs) (*32*), and ancient human selective sweep regions (Supplemental File S2 from the reference) (*33*). All annotations were converted to BED format and lifted over to match genome builds when necessary, using the UCSC liftOver tool (*122*). All annotation coordinates and matched regions can be found in our repository: LINK XX

bioRxiv preprint doi: https://doi.org/10.1101/2025.03.07.641231; this version posted February 14, 2026. The copyright holder for this preprint (which was not certified by peer review) is the author/funder, who has granted bioRxiv a license to display the preprint in perpetuity. It is made available under aCC-BY 4.0 International license.

ES-PGS replication in SPARK

To validate our ES-PGS results in a separate sample we used the SPARK cohort, a large genetic study of individuals with autism and their family members (*36*). For the replication, we utilized imputed SNP array data that we have previously described (*123*) to compute an ES-PGS using the same workflow as in our EpiSLI sample. The self-reported language and psychiatric diagnosis phenotypes, came from a questionnaire as part of a previously described research match where we had > 1,000 adults with autism or parents of children with autism complete an online language battery (*124*). We grouped all self-reported language-related diagnoses (language impairment, dyslexia, hearing impairment, stuttering, and speech impediments), and psychiatric diagnoses together (major depression, generalized anxiety disorder, bipolar, schizophrenia, substance abuse, OCD, and Tourette's). Given the large number of people without any language or psychiatric diagnosis, we used zero inflated Poisson regression models to determine the relationship of the HAQER CP-PGS with having any language or psychiatric diagnosis, as well as the number of language or psychiatric diagnoses. We used the log-ratio test to determine if adding the HAQER CP-PGS term significantly improved prediction accuracy in the zero inflated Poisson regression models. All other phenotypes came from the SPARK December 2014 phenotype release. All analyses included age, sex, background CP-PGS, HAQER matched control CP-PGS, and the first 5 genetic principal components as covariates.

SPARK rare ancestral reversion analysis

To explore the effects of rare genetic variation in evolutionary significant regions on language ability, we used the whole genome sequencing data from the previously described SPARK cohort (max N = 11,545) (*36*). Briefly, we merged the whole genome sequencing data provided by SPARK (WGS batches 1-4) using bcftools, then used the same processing pipeline as we did for the EpiSLI cohort: filtered to variants with a QUAL and individual quality score ≥ 20, and annotated the variants with VEP (v109) (*83*). To identify rare variants we then filtered variants in both datasets to have a maximal reference population allele frequency < 1% and an allele frequency < 1% in SPARK. Ancestral alleles were identified using those provided in the original HAQER manuscript (*34*). We identified all reversion variants within 10Kb of HAQERs from the updated set (*31*), HARs (*32*),

bioRxiv preprint doi: https://doi.org/10.1101/2025.03.07.641231; this version posted February 14, 2026. The copyright holder for this preprint (which was not certified by peer review) is the author/funder, who has granted bioRxiv a license to display the preprint in perpetuity. It is made available under aCC-BY 4.0 International license.

or random non-coding (RAND) (*34*) sequence and counted the number of rare ancestral reversions each sample had in each of these elements. We removed outlier samples who had > 2.5 median absolute deviations away from the median value for either HAQER, HAR, or random reversions, but had consistent phenotypic associations even when including these outliers in the analysis. We then used these reversion counts for association with speech and language phenotypes in SPARK.

Transcription Factor Analysis

Variant selection and annotation

We analyzed transcription factor binding sites in three distinct genomic contexts: Human Accelerated Regions (HARs), Human-Accelerated Quickly Evolved Regions (HAQERs), and matched random genomic regions (RAND, taken from (*31, 34*)). Position weight matrices (PWMs) for 633 human transcription factors were obtained from the JASPAR2020 database (*63*), with pseudocounts adjusted according to base frequency distributions.

Variants were filtered using strict quality control criteria as described above. For this analysis, we retained only biallelic single nucleotide variants (SNVs) located within feature boundaries that exhibited minor allele frequencies below 1% across all reference populations provided by VEP (*83*) and a minor allele frequency below 5% in our smaller EpiSLI sample. Complete great ape allele information was required for each variant, including data from the human reference (hg19), Neanderthals and Denisovans, Chimpanzee, Bonobo, Gorilla, and Orangutan genomes (*34, 125–128*). Briefly, archaic hominin genotype data came from VCFs with genotypes for the three high coverage Neanderthals (Altai (*125*), Chagyrskaya (*126*), and Vindija (*127*)) and one Denisovan (*128*) produced from high-coverage while genome sequencing were downloaded from the Max Planck Institute for Evolutionary Anthropology website and merged with our data. The final dataset comprised genotype information from 15,729 rare variant sites across 350 individuals, with corresponding variant annotations.

Reversion Status Determination

To characterize the evolutionary trajectory of variants, we developed a machine learning approach to impute hominin (human reference and most common allele from Neanderthal and Denisovan

bioRxiv preprint doi: https://doi.org/10.1101/2025.03.07.641231; this version posted February 14, 2026. The copyright holder for this preprint (which was not certified by peer review) is the author/funder, who has granted bioRxiv a license to display the preprint in perpetuity. It is made available under aCC-BY 4.0 International license.

genomes matched) to human-chimp ancestral reversion status where direct determination was not available. We implemented an elastic net regression model (α = 0.9) using the glmnet package in R (*129*), incorporating great ape allele states as predictors of reversion status as given in (*34*). To address class imbalance, we applied weights to ensure equal representation across sequence context types (HAQER, HAR, random). Reversion status was assigned using a probability threshold of 0.95 (i.e., at least a 95% predicted probability of being a human-chimp ancestral reversion), with known states preserved for training data.

Sequence and Motif Analysis

For each variant, we extracted 51-base pair genomic windows centered on the variant position from the human reference genome (hg19). Alternative sequences were generated by substituting variant alleles into the reference background. We then calculated maximal motif scores for both reference and alternative sequences across all JASPAR2020 human transcription factor motifs. Scores were computed on both forward and reverse complement strands, with the maximum score retained for reference and alternate alleles of each variant-motif pair.

Language Association Analysis

Core language ability was assessed using the F1 measure as described above in the factor analysis. We computed burden scores for each transcription factor motif by combining variant effects weighted by genotype status of reversion sites. Linear regression models were used to estimate the associations between individual context-specific (aggregate) motif scores and language ability.

Transcription Factor Motif Score Selection Analysis

For each transcription factor, the (Z-scaled) difference in reference and alternate allele motif scores was modeled as a linear function of variant sequence context (HAQER, HAR, or random) and a binary reversion status indicator. Separate reversion effects were estimated for HAR, HAQER, and random region variants as a sequence context by reversion interaction term. Estimated beta coefficients from these terms, as well as their standard errors, were extracted for use in downstream analyses.

bioRxiv preprint doi: https://doi.org/10.1101/2025.03.07.641231; this version posted February 14, 2026. The copyright holder for this preprint (which was not certified by peer review) is the author/funder, who has granted bioRxiv a license to display the preprint in perpetuity. It is made available under aCC-BY 4.0 International license.

Joint Selection-Language Enrichment Analysis

We used York regression analysis (*130*) to examine relationships between hominin-divergent motif integrity and language-related effects. This approach accounts for uncertainty in both variables (i.e., language association betas and hominin-divergent binding effect betas). Prior to the analysis, the sign of the hominin-divergent binding binding betas were flipped such that positive values indicate human-gained motif integrity scores (i.e., when reversions to the human-chimp ancestral allele tend to decrease motif scores). York regression betas, their standard errors, a Chi-squared goodness of fit statistic and its p-value were extracted to interpret the significance of the overall relationship between TF motif integrity (i.e., motif score) and motif score effect on individual differences in language ability, for each sequence context. Individual TF motifs of interest are those with nominal significance ($p < 0.05$) for both selection for motif integrity and for positive association of aggregate motif integrity with higher F1 core language scores. Transcription factors were classified into families using InterPro annotations (*131*) of representative families found to be significantly associated with a reversion effect in either direction (using www.string-db.org) (*132*). To identify transcription factor families showing convergent patterns of hominin selection and language association, we performed 2x2 Fisher's exact tests comparing the proportion of motifs with concordant effects (hominin gained binding AND positive language association vs. all other combinations) across families. Odds ratios with 95% confidence intervals and p-values were computed for each TF family.

Ancient DNA

DNA data and sample age information for ancient *Homo sapiens* came from the Allen Ancient DNA Resource (AADR) version 54. We downloaded the publicly available EIGENSTRAT formatted files and converted them to PLINK format using the EIGENSOFT tool. We then merged the ancient genomes with our EpiSLI and 1000 Genomes Europeans dataset to ensure we were using comparable SNPs for our ES-PGS selection analysis as we did in our discovery sample. We identified ancient west Eurasians using the same criteria as a recent large-scale selection analysis (*133*). Briefly, we filtered to samples found between longitude 25W and 60E and latitude 35N to 80N, samples passing quality control with an assessment labeled as "PASS", and sample ages > 0 but < 20,000 years old.

bioRxiv preprint doi: https://doi.org/10.1101/2025.03.07.641231; this version posted February 14, 2026. The copyright holder for this preprint (which was not certified by peer review) is the author/funder, who has granted bioRxiv a license to display the preprint in perpetuity. It is made available under aCC-BY 4.0 International license.

We then computed ES-PGS for CP in HAQERs using the same methodology as we did in the EpiSLI, SPARK, and ABCD samples for use in our polygenic selection analysis. Given the challenges of accounting for population structure in ancient DNA, we opted to use a LMM based approach instead of a traditional PC based approach as recommended by Akbari et al., 2024 (*133*). With the ancient west Eurasian subsample, we then identified independent SNPs using 1000 Genomes as the LD reference with PLINK's "–indep-pairwise" function (window size = 1000bp, step size = 1bp, r^2 threshold = 0.05, MAF ≥ 5%) (*134*). Next, we identified samples and SNPs with low missingness for GRM calculation (samples missing < 50% of independent SNPs, and SNPs missing in < 10% of those samples). Finally, we computed the genetic relatedness matrix (GRM) with GCTA (*135*) with the QC passing samples and SNPs and removed duplicate/twin samples for subsequent analysis (GCTA "grm-cutoff" of 0.9).

We then used the 3,244 QC passing samples and the GRM based on the 12,146 QC passing SNPs for the ES-PGS analysis. We implemented the LMM based polygenic selection analysis with the `gaston` (*136*) R package (`lmm.aireml` function), allowing us to account for the GRM which reflects population structure and relatedness of the sample. We used log10(sample age) as the outcome variable and HAQER CP-PGS and background CP-PGS as the independent variables (similar to the ES-PGS analysis we used in our other samples).

Additionally, we computed HAQER CP-PGS in the 10 available that are part of this release of the AADR. We compared these archaic hominins to ancient humans (described above), and 1000 Genomes Europeans, all data was processed together using the same set of SNPs to ensure polygenic scores would be comparable.

Detecting balancing selection in modern genomes

To detect signatures of balancing selection, we analyzed the WGS data we generated in EpiSLI. First, we subset to regions of interest (HAQERs, HARs, RAND, and HAQER matched control sequences). Then we computed a site frequency spectrum (SFS) for each sequence class, calculating the proportion of variants in each minor allele frequency bin. We compared HAQERs SFS to the SFS of HARs, RAND, and matched control sequences, to identify whether HAQERs had a relative enrichment of intermediate frequency variants -which can indicate balancing selection (or ongoing selection). To create 95% confidence intervals for HAQERs we generated 1,000 random bootstrap

bioRxiv preprint doi: https://doi.org/10.1101/2025.03.07.641231; this version posted February 14, 2026. The copyright holder for this preprint (which was not certified by peer review) is the author/funder, who has granted bioRxiv a license to display the preprint in perpetuity. It is made available under aCC-BY 4.0 International license.

samples of HAQERs (sampled with replacement) and computed the allele frequency proportions within in each sampled HAQER set, These confidence intervals allowed us to more confidently determine whether there was enrichment or depletion across MAF bins in HAQERs when compared to other sequence types.

Next, to more formally test our balancing selection hypothesis we identified common independent SNPs in these regions using PLINK (*134*), using the ”–indep-pairwise” function (window size = 200bp, step size = 50bp, r^2 threshold = 0.5, MAF ≥ 5%) (*134*). This identified common independent SNPs in HAQERs, HARs, RAND sequences. We then computed individual level method of moments F-coefficients for each class of variation using the ”–het” function in PLINK (*134*), which derives statistics based on the expected number of homozygotes and observed homozygotes (with more negative values indicating there are more heterozygotes than expected). We compared values between classes using paired t-tests to determine if there was excess heterozygosity in HAQERs, a signature of balancing selection.

ES-PGS analysis in ABCD

To explore the effects of HAQER CP-PGS in prenatal development we analyzed the ABCD cohort, a large longitudinal study of adolescent development with genetic, cognitive, brain imaging, and developmental phenotypes (*137*). All phenotypes used in ABCD came from the v4.0 data release. Similar to the SPARK replication analysis, we utilized imputed SNP array data that we have previously described (*123*) to compute ES-PGS using the same workflow as in our EpiSLI and SPARK samples. We computed genetic relatedness using GCTA (*135*) in the merged ABCD and SPARK dataset. Using the relatedness matrix, we identified unrelated individuals for ES-PGS analysis with brain imaging, cognitive, and birth phenotypes (genetic relatedness < 0.05). All evolutionary stratified polygenic scores were adjusted for genetic principal components, similar to the SPARK and EpiSLI cohorts. We included age, sex, and genetic principal components as covariates in the ES-PGS analyses.

To investigate potential evolutionary trade-offs underlying the balancing selection observed in HAQERs, we examined the relationship between HAQER CP-PGS and both cognitive performance and birth complications in ABCD. We employed a two-stage approach to address confounding and maximize signal detection. First, we applied a modified Remove Unwanted Variation using

bioRxiv preprint doi: https://doi.org/10.1101/2025.03.07.641231; this version posted February 14, 2026. The copyright holder for this preprint (which was not certified by peer review) is the author/funder, who has granted bioRxiv a license to display the preprint in perpetuity. It is made available under aCC-BY 4.0 International license.

residuals (RUVr) approach (*138*) to remove variance components in the phenotypic data that were orthogonal to the HAQER CP-PGS signal. Specifically, we identified and removed k = 15 principal components from the phenotype matrix that captured variance attributable to measured confounders (age, sex, ancestral background from genetic principal components) and unmeasured technical or biological factors not associated with HAQER CP-PGS. This denoising step preserved only phenotypic variance potentially related to our polygenic score of interest while removing confounding sources of variation. Second, we performed canonical correlation analysis (CCA) (*139*) on the RUVr-adjusted phenotypes to identify composite phenotypic dimensions that maximally correlated with HAQER CP-PGS. The CCA simultaneously analyzed two phenotypic domains (each having a block): cognitive performance measures (including memory, executive function, and processing speed tasks) and birth complication-related variables (including cesarean section delivery, intracranial volume measured from MRIs, and other obstetric risk factors). Sparsity constraints ($\lambda = 0.75$) were applied to the phenotypic loadings to improve interpretability. While we present correlation statistics for completeness, we acknowledge these may be inflated due to the optimization procedure; our primary inference focuses on the qualitative pattern of associations rather than specific effect sizes.

HAQER enrichment analysis

To determine enrichment between loci of interest (prenatal scQTLs, birth head circumference GWAS loci, and mammalian vocal learning enhancers) with our evolutionary regions of interest (HAQERs, HARs, and random sequence), we used the ”intervalOverlap” function from gonomics (*140*). This allowed us to compute expected versus actual overlaps (based on size of the human genome and the provided annotations) providing an enrichment p-value for each region.

Evolutionary annotations

Given the two sets of HAQER annotations available (*31, 34*), created using different reference genomes and sequencing methodology we opted to use a conservative set of HAQERs for enrichment analyses to ensure any associations were not due to artifacts. The conservative set of HAQERs was identified by overlapping regions in the two separate HAQER sets using the bedtools ”intersect” function (*120*), leaving nearly 900 overlapping HAQER regions. We included the set of HARs and

bioRxiv preprint doi: https://doi.org/10.1101/2025.03.07.641231; this version posted February 14, 2026. The copyright holder for this preprint (which was not certified by peer review) is the author/funder, who has granted bioRxiv a license to display the preprint in perpetuity. It is made available under aCC-BY 4.0 International license.

random sequence (matched to basic biological properties in HAQERs like size and non-coding sequence) from the original HAQER paper (*34*) for all enrichment analyses, allowing us to directly compare annotations. Similar to the other genomic analyses, we restricted analysis of all annotations to autosomal regions.

Defining human-specific and cell-type specific chromatin accessible regions

To identify whether HAQERs provided humans with novel regulatory mechanisms in specific neural cell-types, we examined data from snATAC-seq of human and mouse brains (*38*). To identify human-specific and cell-type specific cCREs, we used Suppelemental Table 6 provided by the authors to get hg38 coordinates of cCREs and filtered to ”humanSpec” cCREs using data provided in Supplementary Table 20. Similarly, to identify human-mouse conserved cCREs, we used Suppelemental Table 6 provided by the authors and filtered to ”CA_cons” cCREs using data provided in Supplementary Table 20. We added a 100Kb flank around all chromatin accessible regions and merged overlapping regions using the bedtools ”merge” function, allowing us to test whether evolutionary annotations are enriched around cell-type specific chromatin accessibility regions.

Defining neurodevelopmental scQTLs

To determine if HAQERs, HARs, and RAND sequences significantly influence gene expression in prenatal and postnatal brains, we leveraged scQTLs from two studies. (1) a study of stem cell derived neurons from > 200 individuals, meant to mimic early prenatal cells in the midbrain (*46*) and (2) a study of nearly 400 postmortem adult brains from psychENCODE2 (*48*). For scQTL study (1), we defined scQTLs as all common variants (MAF > 5%) with a p-value $< 5 \times 10^{-4}$, for scQTL study (2) we utilized all provided scQTLs defined as significant by the authors downloaded from `https://psychscreen.wenglab.org/psychscreen/downloads`.

Defining birth head circumference regions

We identified birth head circumference associated genomic loci with a genome-wide suggestive p-value (p-value $< 5 \times 10^{-5}$) and a MAF > 5%, using the largest GWAS available to date (*49*).

bioRxiv preprint doi: https://doi.org/10.1101/2025.03.07.641231; this version posted February 14, 2026. The copyright holder for this preprint (which was not certified by peer review) is the author/funder, who has granted bioRxiv a license to display the preprint in perpetuity. It is made available under aCC-BY 4.0 International license.

Given the influence of LD on human traits, we added a 100Kb flank around all associated SNPs and merged overlapping regions using the bedtools ”merge” function, allowing us to test whether evolutionary annotations are enriched around associated SNPs.

Defining mammalian vocal learning enhancer regions

We gathered previously established mammalian vocal learning enhancer regions from a recent cross-species analysis (*15*), which identified 50 open chromatin regions in the motor cortex strongly associated with vocal learning. Since these regions were provided in mouse genome coordinates (mm10), we lifted these over to hg19 and we added 1Mb flanks around these enhancer regions which were then used to test whether evolutionary annotations are enriched near established vocal learning enhancers.

Convergent evolution of vocal learning analysis

To test for evidence of convergent evolution of ”HAQER-like” sequences in vocal learning species, we utilized a large dataset of > 400 species with whole genome data aligned to the human reference genome (hg38) (*52*). We parsed the alignments with Biopython (*141*) to subset to regions of interest. For each species and sequence type (HAQERs or HARs), we computed a sequence similarity (using the number of bases matching the human reference genome in these regions). We then used this ”HAQER-like” sequence similarity to predict vocal learning status in 170 non-primate species that have been previously described (*15*). To determine statistical significance and account for species relatedness we used phylogenetic logistic regression (*53*) with the `phylolm` package (*54*) in R. We did similar analyses for brain size and birth:adult weight ratio using species level phenotype data from the PanTHERIA dataset (*64*).

Statistical analysis

All statistical analysis was done in R (version 4.3.1) (*73*).

bioRxiv preprint doi: https://doi.org/10.1101/2025.03.07.641231; this version posted February 14, 2026. The copyright holder for this preprint (which was not certified by peer review) is the author/funder, who has granted bioRxiv a license to display the preprint in perpetuity. It is made available under aCC-BY 4.0 International license.

Approach to multiple testing correction and triangulating evidence

To address multiple testing concerns, we employed False Discovery Rate (FDR) correction for analyses testing more than 20 hypotheses, allowing us to limit type 1 errors (false positives). This was applied to the EpiSLI CBCL mental health score analysis, genome-wide PGS analysis, and the ES-PGS analysis where we conducted many statistical tests.

We adopted a comprehensive approach to validate our key findings through multiple lines of evidence. We established confidence in our results through: (1) replication across independent cohorts (e.g., EpiSLI, SPARK, and ABCD); (2) identifying convergent evidence across different analytical approaches (e.g., common variant, rare variant, and transcription factor binding analyses); (3) demonstration of specificity, showing HAQERs' associations with language but not nonverbal IQ; (4) evolutionary support from ancient DNA and cross-species analyses; and (5) mechanistic support through analysis of transcription factor binding, enrichment for human-specific and cell-type specific chromatin accessible regions, and enrichment of variants influencing prenatal gene regulation. This multi-faceted approach allowed us to distinguish robust biological signals from statistical noise, even when some individual analyses showed moderate statistical significance. Our findings' consistency across diverse data types, species, and analytical methods provides stronger evidence than would be achieved through any single statistical test, regardless of its p-value.

Supplementary Figures

bioRxiv preprint doi: https://doi.org/10.1101/2025.03.07.641231; this version posted February 14, 2026. The copyright holder for this preprint (which was not certified by peer review) is the author/funder, who has granted bioRxiv a license to display the preprint in perpetuity. It is made available under aCC-BY 4.0 International license.

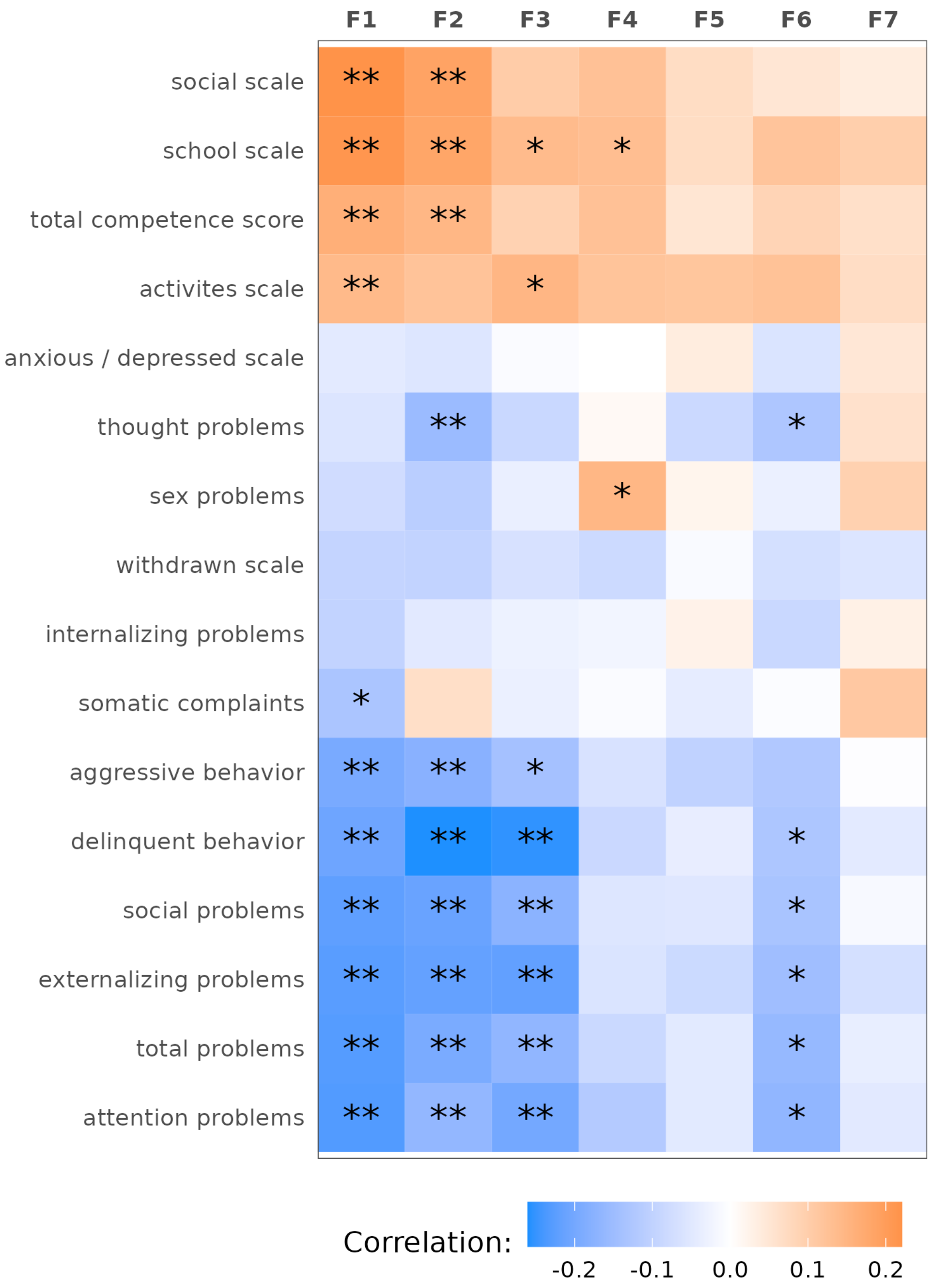

Figure S1: **Mental health correlates of EpiSLI factors**

Heatmap of Pearson correlation coefficients (*r*) of CBCL scales with EpiSLI factor scores (N = 241 individuals). Color indicates the association between that evolutionary annotation and the EpiSLI factor score (orange = positive correlation, blue = negative correlation. "**" indicates FDR < 0.05, and "*" indicates unadjusted p-value < 0.05.

bioRxiv preprint doi: https://doi.org/10.1101/2025.03.07.641231; this version posted February 14, 2026. The copyright holder for this preprint (which was not certified by peer review) is the author/funder, who has granted bioRxiv a license to display the preprint in perpetuity. It is made available under aCC-BY 4.0 International license.

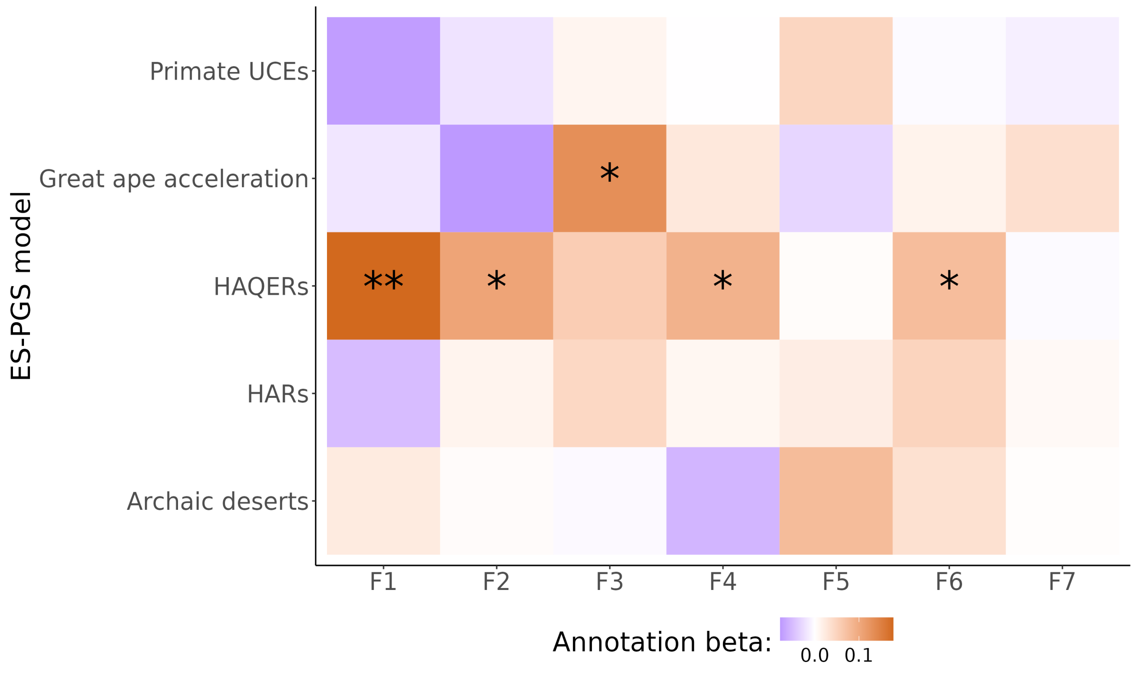

Figure S2: **Evolutionary history of EpiSLI factors**

Heatmap of ES-PGS β's stratifying the CP-PGS based on evolutionary annotations. Color indicates the association between that evolutionary annotation and the EpiSLI factor score (orange = positive ES-PGS β, purple = negative ES-PGS β). "**" indicates FDR < 0.01, and "*" indicates unadjusted p-value < 0.05.

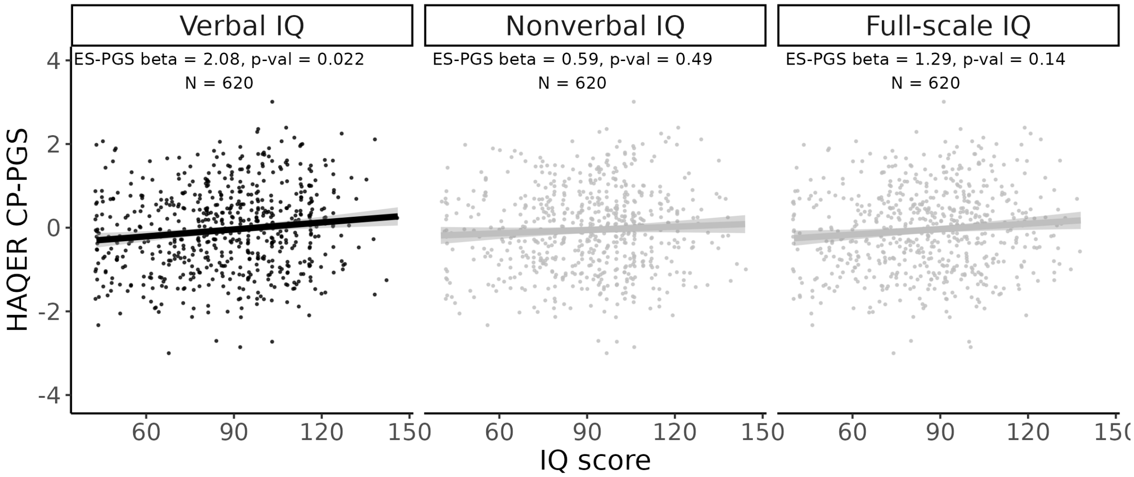

Figure S3:

HAQER CP-PGS is associated with verbal IQ in an independent cohort

ES-PGS results of HAQER CP-PGS and clinical IQ assessment scores from the SPARK dataset (N = 620).

bioRxiv preprint doi: https://doi.org/10.1101/2025.03.07.641231; this version posted February 14, 2026. The copyright holder for this preprint (which was not certified by peer review) is the author/funder, who has granted bioRxiv a license to display the preprint in perpetuity. It is made available under aCC-BY 4.0 International license.

Figure S4: **ES-PGS differences across human history**

A-C Distribution of HAQER CP-PGS (**A**), background CP-PGS (**B**), or random matched control regions (**C**) across archaic humans (neanderthals and denisovans from the AADR), ancient anatomically modern humans (AADR), and modern Europeans (1000 Genomes dataset). "*" indicates a p-value of < 0.05 from a t-test comparison, and "***" indicates p-value < 0.001.

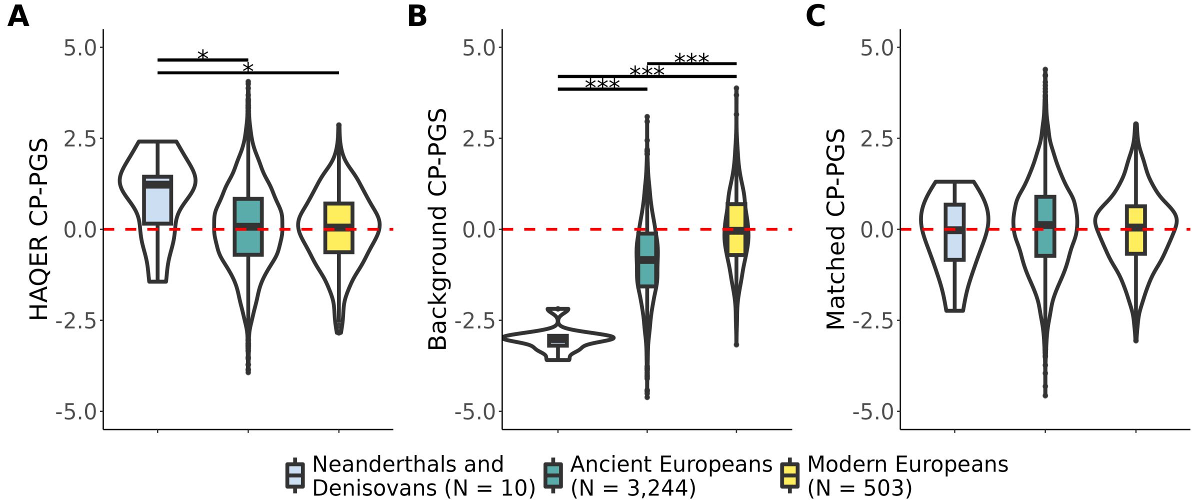

bioRxiv preprint doi: https://doi.org/10.1101/2025.03.07.641231; this version posted February 14, 2026. The copyright holder for this preprint (which was not certified by peer review) is the author/funder, who has granted bioRxiv a license to display the preprint in perpetuity. It is made available under aCC-BY 4.0 International license.

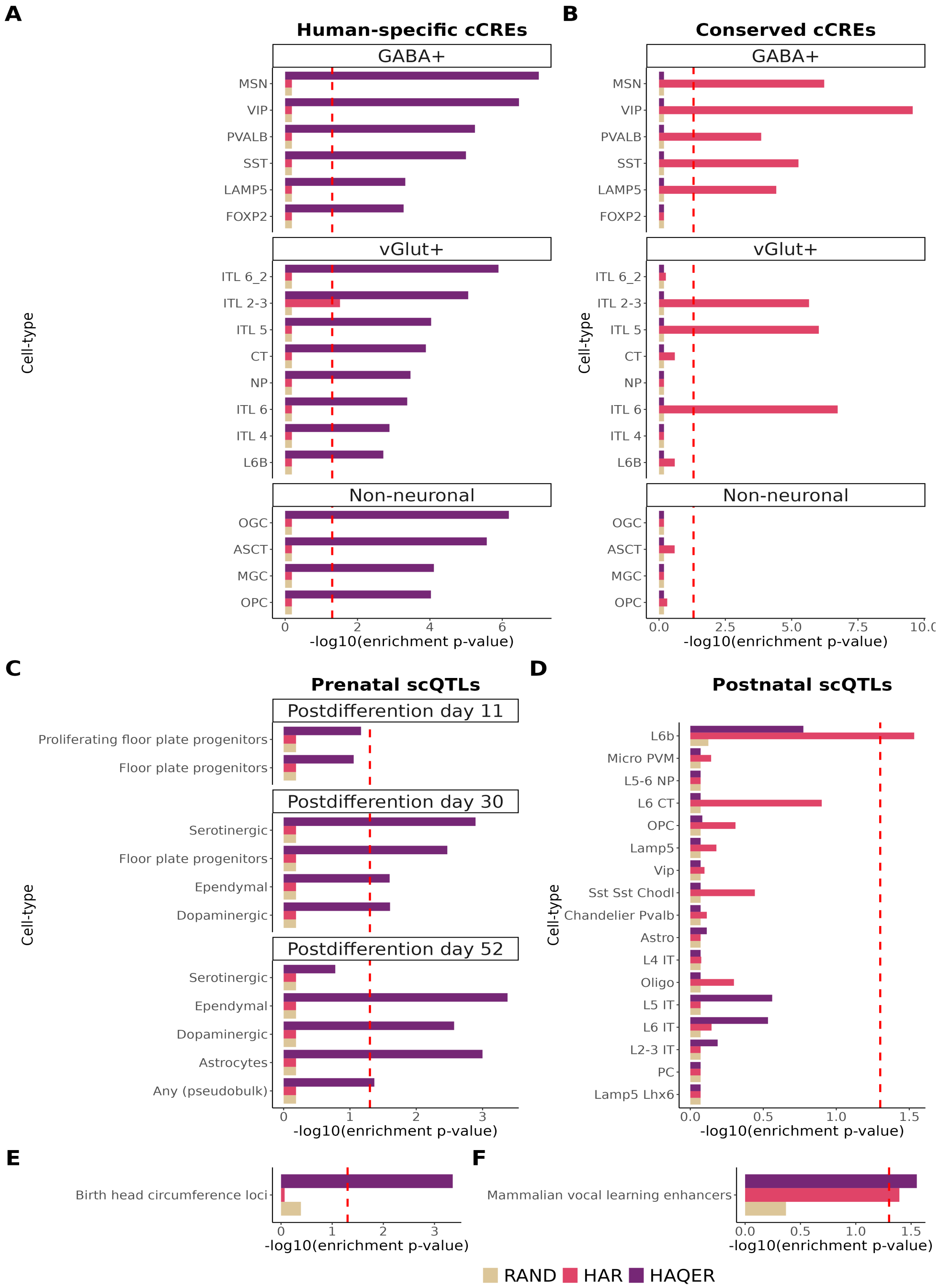

Figure S5: **HAQERs influence prenatal brain development**

A-B Enrichment of HAQERs, HARs, and RAND for brain cCREs from human-specific (**A**) or human-mouse conserved (**B**) elements. **C-D** Enrichment for regulatory variants (scQTLs) across timepoints and cell-types in newly differentiated neurons (**C**) or adult post mortem brains (**D**). **E** Enrichment for common SNPs (MAF > 5%) associated with head circumference at birth. **F** Enrichment for enhancer regions associated with vocal learning across mammals (Wirthlin et al., 2024).
