# Supplementary material for: Rapidly evolved genomic regions shape individual language abilities in present-day humans
Source: bioRxiv. 2025 Mar 10:2025.03.07.641231. Preprint. [Version 1] doi: 10.1101/2025.03.07.641231 (PMC11952349; doi:10.1101/2025.03.07.641231)

bioRxiv preprint doi: https://doi.org/10.1101/2025.03.07.641231; this version posted March 11, 2025. The copyright holder for this preprint (which was not certified by peer review) is the author/funder, who has granted bioRxiv a license to display the preprint in perpetuity. It is made available under aCC-BY 4.0 International license.

15 Supplemental figures

Figure S1: **HAQERs influence prenatal brain development**
a-b Enrichment of HAQERs, HARs, and RAND of prenatal regulatory variants across timepoints and cell-types from newly differentiated neurons (a) or adult post mortem brains (b). **c** HAQER and background CP-PGS associations with total intracranial volume in adolescents (ABCD). **d** HAQER and background CP-PGS associations with total intracranial growth in adolescence (ABCD). **e** Scatterplot comparing how comparing background CP-PGS and birth weight differences between pairs of siblings. x-axis is the residual sibling difference in birth weight after accounting for sibling differences in sex, age, gestation duration, and HAQER CP-PGS. y-axis is the sibling difference in background CP-PGS. **f** Scatterplot comparing how comparing HAQER CP-PGS and birth weight differences between pairs of siblings. x-axis is the residual sibling difference in birth weight after accounting for sibling differences in sex, age, gestation duration, and background CP-PGS. y-axis is the sibling difference in HAQER CP-PGS. **g** Comparing background CP-PGS in sibling pairs where only one is born via c-section. **h** Comparing HAQER CP-PGS in sibling pairs where only one is born via c-section.

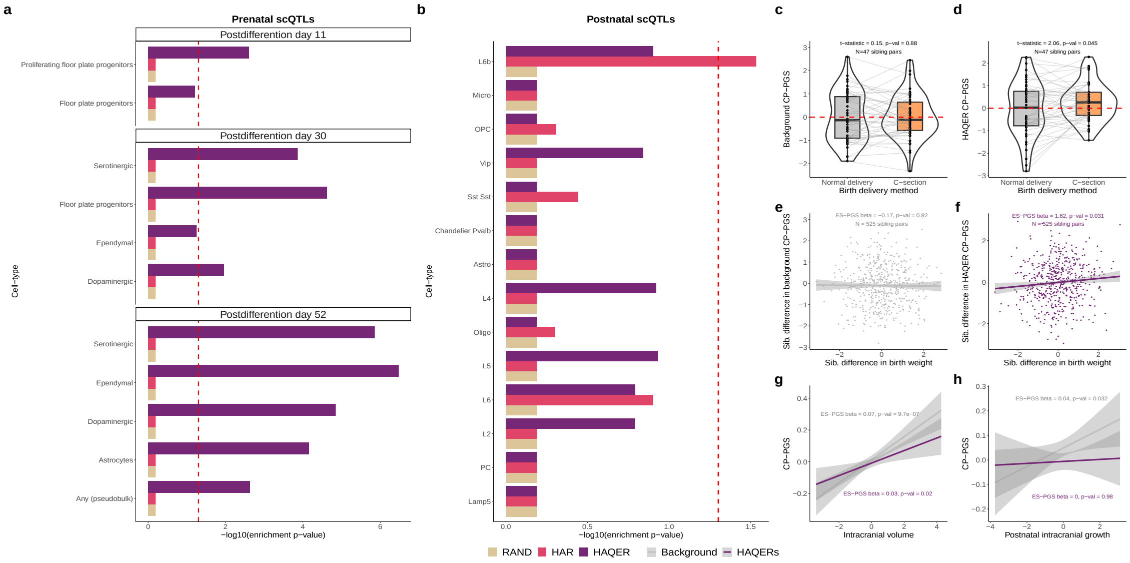

Supplement: Supplementary file 1 [file NIHPP2025.03.07.641231v1-supplement-1.pdf]
